# Terminal and Internal Alkyne Complexes and Azide-Alkyne Cycloaddition Chemistry of Copper(I) Supported by a Fluorinated Bis(pyrazolyl)borate

**DOI:** 10.3390/molecules27010016

**Published:** 2021-12-21

**Authors:** Anurag Noonikara-Poyil, Alvaro Muñoz-Castro, H. V. Rasika Dias

**Affiliations:** 1 Department of Chemistry and Biochemistry, The University of Texas at Arlington, Arlington, TX 76019, USA; anurag.noonikarapoyil@mavs.uta.edu; 2 Grupo de Química Inorgánica y Materiales Moleculares, Facultad de Ingenieria, Universidad Autonoma de Chile, El Llano Subercaseaux 2801, Santiago 8910060, Chile

**Keywords:** copper, acetylene, alkyne, click chemistry, pyrazolyl borate

## Abstract

Copper plays an important role in alkyne coordination chemistry and transformations. This report describes the isolation and full characterization of a thermally stable, copper(I) acetylene complex using a highly fluorinated bis(pyrazolyl)borate ligand support. Details of the related copper(I) complex of HC≡CSiMe_3_ are also reported. They are three-coordinate copper complexes featuring η^2^-bound alkynes. Raman data show significant red-shifts in C≡C stretch of [H_2_B(3,5-(CF_3_)_2_Pz)_2_]Cu(HC≡CH) and [H_2_B(3,5-(CF_3_)_2_Pz)_2_]Cu(HC≡CSiMe_3_) relative to those of the corresponding alkynes. Computational analysis using DFT indicates that the Cu(I) alkyne interaction in these molecules is primarily of the electrostatic character. The π-backbonding is the larger component of the orbital contribution to the interaction. The dinuclear complexes such as Cu_2_(μ-[3,5-(CF_3_)_2_Pz])_2_(HC≡CH)_2_ display similar Cu-alkyne bonding features. The mononuclear [H_2_B(3,5-(CF_3_)_2_Pz)_2_]Cu(NCMe) complex catalyzes [3 + 2] cycloadditions between tolyl azide and a variety of alkynes including acetylene. It is comparatively less effective than the related trinuclear copper catalyst {μ-[3,5-(CF_3_)_2_Pz]Cu}_3_ involving bridging pyrazolates.

## 1. Introduction

Copper is an important metal in alkyne chemistry. It mediates a number of transformations of acetylene as well as larger alkynes including cycloaddition chemistry [1,2,3,4,5], cyclopropenation [6,7,8]. Terminal and Internal Alkyne Complexes and Azide-alkyne Cycloaddition Chemistry of Copper(I) Supported by a Fluorinated Bis(pyrazolyl)borate partial hydrogenation, [9,10] hetero atom-hydrogen bond additions [11,12,13,14,15,16], C_sp_-H bond functionalizations, and alkyne coupling processes [17,18,19,20,21,22,23,24,25,26]. Copper catalyzed azide-alkyne cycloaddition (CuAAC) is perhaps the most popular among the different reaction types due to its virtues of mild reaction conditions, high yields, and regioselectivity, with a major impact on organic and materials chemistry to chemical-biology applications [2,5,27,28,29,30,31,32]. Copper based materials are also useful for the separation of acetylene from CO_2_ and acetylene storage [33,34,35,36,37]. Copper alkyne or alkynide complexes [38] are believed to be key intermediates in many of these reactions. Copper alkynes are used as precursors for the copper deposition as well [39,40,41].

**Figure 1:**
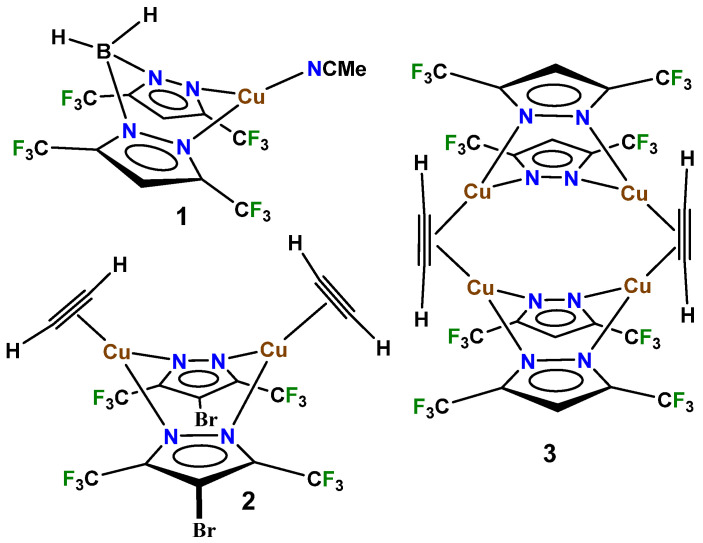
[H_2_B(3,5-(CF_3_)_2_Pz)_2_]Cu(NCMe) (**1**) and a selected group of structurally characterized copper complexes of acetylene, Cu_2_(μ-[4-Br-3,5-(CF_3_)_2_Pz])_2_(HC≡CH)_2_ (**2**) and Cu_4_(μ-[3,5-(CF_3_)_2_Pz])_4_(μ-HC≡CH)_2_ (**3**).

Over the past few years, we have been working on the coordination chemistry [42,43,44,45,46,47] and transformations [6,47,48,49] of alkynes involving copper. For example, we demonstrated that the trinuclear copper(I) pyrazolate {μ-[3,5-(CF_3_)_2_Pz]Cu}_3_ [50] is an effective catalyst for facile azide-alkyne cycloaddition leading to 1,2,3-triazoles, alkyne C(sp)-H bond carboxylation with CO_2_, and S-H addition to alkyne moiety [47]. Some of the alkyne complexes of copper pyrazolates show interesting luminescence [46]. We also discovered that the mononuclear, bis(pyrazolyl)borate [H_2_B(3,5-(CF_3_)_2_Pz)_2_]Cu(NCMe) (**1**, Pz = pyrazolyl; Figure 1) is an excellent mediator of cyclopropenation chemistry of alkynes with ethyl diazoacetate.[6]

In this work, we describe the use of [H_2_B(3,5-(CF_3_)_2_Pz)_2_]Cu(NCMe) as a catalyst in azide-alkyne cycloaddition chemistry between *p*-tolylazide and several alkynes including acetylene and trimethylsilylacetylene. We also describe the isolation of an acetylene complex as well as larger alkyne complexes involving the copper bis(pyrazolyl)borate moiety [H_2_B(3,5-(CF_3_)_2_Pz)_2_]Cu. Such compounds are of current interest [38,51,52] and allow a comparison of mono-nuclear copper bis(pyrazolyl)borates to dinuclear copper(I) pyrazolates (e.g., [H_2_B(3,5-(CF_3_)_2_Pz)_2_]Cu(HC≡CH) (**4**) and Cu_2_(μ-[4-Br-3,5-(CF_3_)_2_Pz])_2_(HC≡CH)_2_ (**2**)). It is also noteworthy that despite the long history of copper(I)-acetylene chemistry [53,54], copper(I) acetylene complexes with detailed structural and spectroscopic data are surprisingly scarce. For example, apart from the dinuclear and tetranuclear copper complexes **2** and Cu_4_(μ-[3,5-(CF_3_)_2_Pz])_4_(μ-HC≡CH)_2_ (**3**) reported by us recently [47], structurally authenticated copper-C_2_H_2_ complexes are limited to [Cu{NH(Py)_2_}(HC≡CH)]BF_4_ and [Cu(phen)(HC≡CH)]ClO_4_ with Cu(η^2^-HC≡CH) moieties [55,56,57] and polymeric or octanuclear, chloride bridged copper(I) adducts containing μ_2_−η^2^,η^2^-(HC≡CH) moieties [58,59,60].

## 2. Results and Discussion

**Scheme 1:**
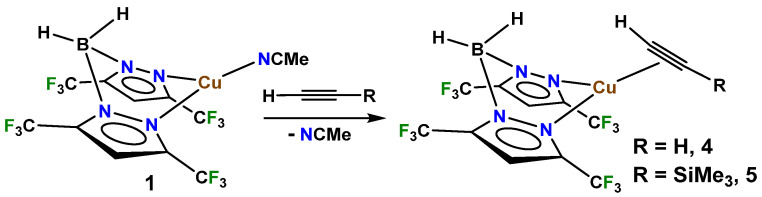
Synthesis of [H_2_B(3,5-(CF_3_)_2_Pz)_2_]Cu(HC≡CH) (**4**) and [H_2_B(3,5-(CF_3_)_2_Pz)_2_]Cu(HC≡CSiMe_3_) (**5**) from [H_2_B(3,5-(CF_3_)_2_Pz)_2_]Cu(NCMe) (**1**) and the corresponding alkyne.

The bis(pyrazolyl)borate copper(I) complex [H_2_B(3,5-(CF_3_)_2_Pz)_2_]Cu(NCMe)[42] reacts with purified acetylene (~1 atm) [61,62] in CH_2_Cl_2_, affording [H_2_B(3,5-(CF_3_)_2_Pz)_2_]Cu(HC≡CH) (**4**) as a white solid in >90% yield (Scheme 1), which is quite amenable to detailed spectroscopic and structural studies. The room temperature ^1^H NMR spectrum of **4** in CDCl_3_ displayed the acetylenic proton resonances at δ 4.70 ppm. That is a significant downfield shift relative to the corresponding signal of the free acetylene (δ 2.01 ppm) [63]. The ^13^C resonance of the acetylenic carbons appears at δ 80.2 ppm, which is a downfield shift of 7.0 ppm relative to that of the free acetylene (δ 73.2 ppm) [63]. The ῡ_C__≡C_ band of solid **4** in the Raman spectrum was observed at 1819 cm^−1^, representing a 155 cm^−1^ red shift relative to the corresponding stretching frequency of the free HC≡CH (1974 cm^−1^) [64]. This red shift is not as high as that observed for Cu_4_(μ-[3,5-(CF_3_)_2_Pz])_4_(μ-HC≡CH)_2_ (**3** with ῡ_C__≡C_ of 1638 cm^−1^) containing a μ_2_−η^2^,η^2^-(HC≡CH) (which is a formally 4e-donor, bridging acetylene). Table 1 shows available, albeit limited, ^1^H and ^13^C NMR data and C≡C stretch of structurally characterized copper complexes featuring a formally 2e-donor η^2^-(HC≡CH). [H_2_B(3,5-(CF_3_)_2_Pz)_2_]Cu(HC≡CH) (**4**) shows the smallest downfield shift of the acetylenic NMR signal, and red-shift of C≡C stretching frequency relative to that of the free HC≡CH among these (although the differences are minor), suggesting relatively weaker σ/π-interaction between the copper(I) and acetylene ligand in terms of the Dewar-Chatt-Duncanson picture [65,66]. A rare, well-authenticated silver(I)-acetylene complex, [HB(3,5-(CF_3_)_2_Pz)_3_]Ag(HC≡CH) is also available for a comparison (although it has a *tris(pyrazolyl)borate* (not bis(pyrazolyl)borate) ligand support) [67]. It is a system that features relatively low M→alkyne backbonding. The acetylenic ^1^H signal of this silver(I) complex has been observed at δ 3.48 ppm, which is an even smaller downfield shift from the free acetylene resonance (δ 2.01 ppm), compared to that observed for [H_2_B(3,5-(CF_3_)_2_Pz)_2_]Cu(HC≡CH) (**4**). Interestingly, alkyne resonance of [HB(3,5-(CF_3_)_2_Pz)_3_]Ag(HC≡CH) in ^13^C NMR appears at δ 66.3 ppm. 

Overall, acetylenic ^1^H and ^13^C resonance of d-block metal complexes bearing 2e-donor, η^2^-acetylene ligands has been observed (keeping in mind that η^2^-acetylene can also serve as a formally 4e-donor moiety) at quite a wide chemical shift range [68]. For example, NMR spectra of Ru(II) complex [Cp*Ru(HC≡CH)(PEt_3_)_2_][BPh_4_] [69] and Ni(0) complex (Ph_3_P)_2_Ni(HC≡CH) [70] display their signals for the metal bound C_2_H_2_ in ^1^H and ^13^C at δ 4.38, 66.14 ppm and δ 6.41, 122 ppm, respectively. The latter nickel complex is expected to display more pronounced M→alkyne backbonding than in **4**. For comparison, protons of the *bridging* acetylene group of Cu_4_(μ-[3,5-(CF_3_)_2_Pz])_4_(μ-HC≡CH)_2_ (**3**) have been observed at δ 6.16 ppm [47].

**Figure 2:**
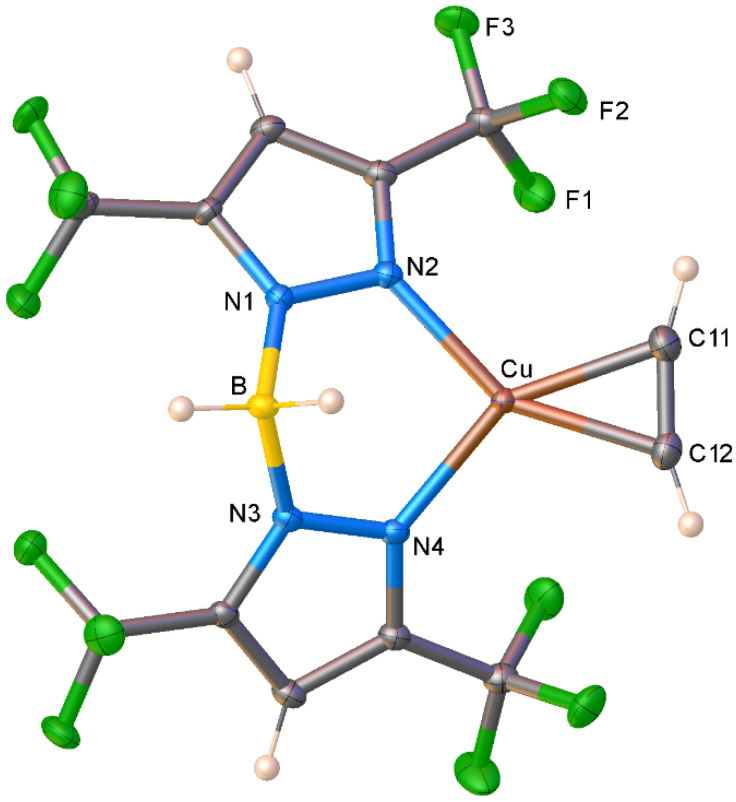
Molecular structure of [H_2_B(3,5-(CF_3_)_2_Pz)_2_]Cu(HC≡CH) (**4**); ORTEP view with 50% probability ellipsoids are shown. Selected bond distances (Å) and angles (°): Cu-N2 1.981(3), Cu-N4 1.981(3), Cu-C11 1.972(3), Cu-C12 1.973(3), C11-C12 1.225(5), B-N1 1.570(4), B-N3 1.569(4), Cu···B 3.046, C11-Cu-C12 36.17(14), N2-Cu-N4 96.63(10), N4-Cu-C12 113.19(13), N2-Cu-C11 114.01(13), N1-B-N3 107.6(2).

The X-ray crystal structure of [H_2_B(3,5-(CF_3_)_2_Pz)_2_]Cu(HC≡CH) (**4**) is illustrated in Figure 2. It is a three coordinate, trigonal planar copper-acetylene complex. The acetylene ligand is oriented parallel to the NCuN plane so as to maximize back-bonding interactions [73]. Selected bond distances and angles of **4** and copper complexes featuring 2e-donor, η^2^-acetylene ligand in the literature are given in Table 1. The key parameters involving the CuC_2_ core are remarkably similar between these molecules. This suggests that cationic copper species [Cu{NH(Py)_2_}(HC≡CH)]BF_4_ and [Cu(phen)(HC≡CH)]ClO_4_ featuring relatively electron-rich supporting ligands and neutral copper complexes **4** and **2** involving weakly donating fluorinated ligands have similar effects on the Cu-C_2_H_2_ alkyne moiety, or produce effects that are not large enough to be parsed out by routine X-ray crystallography. They both show slightly elongated C≡C bonds relative to the free acetylene (1.181(7) Å) [74], but these changes are overshadowed by the somewhat high esd associated with bond distance measurements. 

In addition to acetylene, we also tested the use of HC≡CSiMe_3_ as a substrate in CuACC chemistry. Considering that structurally authenticated metal complexes of η^2^-HC≡CSiMe_3_ are rare (a search of Cambridge Structural Database [75] disclosed only three such examples involving transition metal ions) [76,77,78] and unknown for copper to our knowledge [75], we also synthesized [H_2_B(3,5-(CF_3_)_2_Pz)_2_]Cu(HC≡CSiMe_3_) (**5**) for a detailed study. Treatment of [H_2_B(3,5-(CF_3_)_2_Pz)_2_]Cu(NCMe) with HC≡CSiMe_3_ in CH_2_Cl_2_ led to **5** in 91% yield (Scheme 1). It is a white solid and was characterized by NMR and Raman spectroscopy and X-ray crystallography. The ῡ_C__≡C_ band of solid **5** in the Raman spectrum was observed at 1870 cm^−1^, which is a 237 cm^−1^ red shift relative to the corresponding stretching frequency of the free HC≡CSiMe_3_ (2107 cm^−1^). This ῡ(C≡C) is similar to that reported for [HC{C(CF_3_)CO}_2_]Cu(HC≡CSiMe_3_) [41]. This suggests the presence of an η^2^-HC≡CSiMe_3_ bound alkyne moiety on copper(I) [46,51].

**Figure 3:**
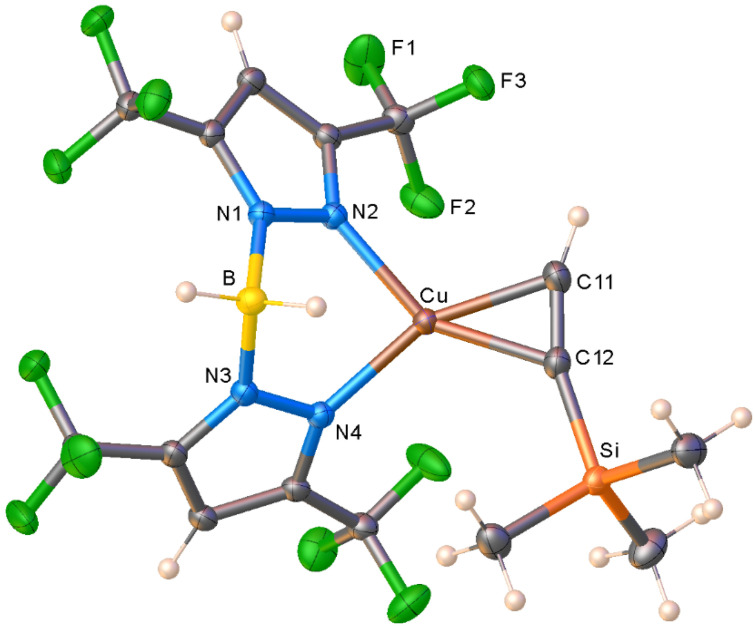
Molecular structure of [H_2_B(3,5-(CF_3_)_2_Pz)_2_]Cu(HC≡CSiMe_3_) (**5**); ORTEP view with 50% probability ellipsoids are shown. Selected bond distances (Å) and angles (°): Cu-N2 1.9857(9), Cu-N4 1.9845(9), Cu-C11 1.9600(12), Cu-C12 1.9957(11), C11-C12 1.2343(17), B-N1 1.5778(15), B-N3 1.5737(16), C12-Si 1.8713(12), Cu···B 2.964, C11-Cu-C12 36.35(5), N2-Cu-N4 90.59(4), N4-Cu-C12 120.05(4), N2-Cu-C11 112.92(5), N1-B-N3 106.41(9), C11-C12-Si 160.64(11).

X-ray crystal structure of [H_2_B(3,5-(CF_3_)_2_Pz)_2_]Cu(HC≡CSiMe_3_) (**5**) is depicted in Figure 3. It is a monomeric, trigonal planar copper complex with an η^2^-HC≡CSiMe_3_ bound alkyne moiety. The HC≡CSiMe_3_ is bonded slightly asymmetrically as evident from the marginally longer Cu-C12, which is a carbon atom with the larger, silyl group. The alkyne group shows a significant deviation from the ideal 180° as evident from C≡C-Si angle, 160.64(11)°. This is about 19° bending back of the alkyne group due to the metal ion coordination. As noted above, there are no structural data on related copper η^2^-HC≡CSiMe_3_ complexes for comparisons. The Cp_2_Nb(H)(HC≡CSiMe_3_) [76] and (NMe-Paa)W(CO)F(HC≡CSiMe_3_) (based on a *κ^3^*-[C,N,N′] chelator NMe-Paa = 2-(2-dimethylaminoethyl)methylaminomethylphenyl) [78] complexes are known and display significantly smaller C≡C-Si angles of 141.7(5)° and 138.1(1)° (or much larger deviation from linearity), which points to stronger metal-alkyne σ/π-bonding in these W(II) and Nb(III) complexes, in terms of the Dewar-Chatt-Duncanson model [65,66].

Compounds [H_2_B(3,5-(CF_3_)_2_Pz)_2_]Cu(HC≡CH) (**4**) and Cu_2_(μ-[4-Br-3,5-(CF_3_)_2_Pz])_2_(HC≡CH)_2_ (**2**) (note: non-brominated, Cu_2_(μ-[3,5-(CF_3_)_2_Pz])_2_(HC≡CH)_2_ analog has been observed but not isolated) allow us to compare the effects of replacing a [H_2_B]^+^ with a [(alkyne)Cu]^+^ moiety in these systems. Although structural features of N_2_CuC_2_ core are very comparable (Table 1), ^1^H and Raman spectroscopic data of the alkyne group suggest that the copper site in the mononuclear **4** is slightly more Lewis acidic than that of dinuclear **5**, despite having a 4-bromo pyrazolate in the latter. Table S1 (Supplementary Materials) in supporting information shows two additional sets of molecules, more closely related to each other; [H_2_B(3,5-(CF_3_)_2_Pz)_2_]Cu(EtC≡CEt) (**6**) [6] and Cu_2_(μ-[3,5-(CF_3_)_2_Pz])_2_(EtC≡CEt)_2_ (**7**)[46] as well as [H_2_B(3,5-(CF_3_)_2_Pz)_2_]Cu(HC≡CPh) (**8**) [42] and Cu_2_(μ-[3,5-(CF_3_)_2_Pz])_2_(HC≡CPh)_2_ (**9**) [47]. To facilitate this analysis, the X-ray crystal structure of [H_2_B(3,5-(CF_3_)_2_Pz)_2_]Cu(EtC≡CEt) was also investigated and the details are included in the Supporting Information [6]. A comparison of mononuclear bis(pyrazolyl)borate systems to the corresponding dinuclear pyrazolate systems show very similar metrical parameters involving the N_2_CuC_2_ cores, except for N-Cu-N angles, which are smaller for the bis(pyrazolyl)borate copper complexes. Closer analysis indicates that this is not because of noticeably longer Cu-N distances in bis(pyrazolyl)borate copper systems but due to their more folded CuN_4_B cores (compared to the flatter CuN_4_C rings in the related dinuclear pyrazolates). This is evident from the separation of the pyrazolyl ring carbons at 4-positions (e.g., Pz-C4···Pz-C4 distance of [H_2_B(3,5-(CF_3_)_2_Pz)_2_]Cu(EtC≡CEt) (**6**) and Cu_2_(μ-[3,5-(CF_3_)_2_Pz])_2_(EtC≡CEt)_2_ (**7**) is 6.18 and 6.38 Å). 

**Figure 4:**
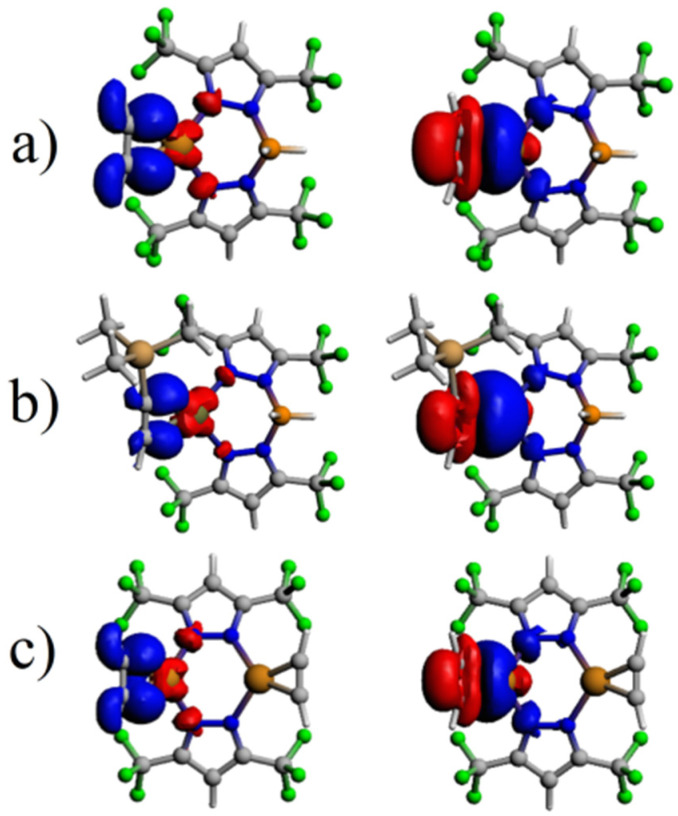
Representative deformation densities accounting for the π-backbonding (left) and σ-donation (right) contribution to the bonding scheme in the formation of alkyl copper complexes (from top to bottom) for (**a**) [H_2_B(3,5-(CF_3_)_2_Pz)_2_]Cu(HC≡CH) (**4**), (**b**) [H_2_B(3,5-(CF_3_)_2_Pz)_2_]Cu(HC≡CSiMe_3_) (**5**) and (**c**) the dinuclear Cu_2_(μ-[3,5-(CF_3_)_2_Pz])_2_(HC≡CH)_2_ (**10**). Charge flow from red to blue.

Further analysis of the alkyne-copper(I) interaction is performed using density functional calculations (Table 2) to understand the variations between the mononuclear and dinuclear species as well as the copper and different alkynes. The overall interaction energy (Δ*E*_int_) between the alkyne and copper(I) center for mononuclear species [H_2_B(3,5-(CF_3_)_2_Pz)_2_]Cu(HC≡CH) (**4**), [H_2_B(3,5-(CF_3_)_2_Pz)_2_]Cu(EtC≡CEt) (**6**), and [H_2_B(3,5-(CF_3_)_2_Pz)_2_]Cu(PhC≡CH) (**8**) is −46.8, −51.5, and −49.4 kcal⸱mol^−1^, respectively, which is further dissected in different contributions within the Ziegler-Rauk energy decomposition analysis (EDA) [79,80]. In this framework, the interaction energy (ΔE_int_) exhibits a larger electrostatic character (ΔE_elstat_) of about ~60% of the stabilizing terms, whereas the orbital contribution to the interaction is about ~35% (ΔE_orb_), with the remaining ~5% attributable to dispersion-type contributions (ΔE_disp_). The ΔE_orb_ involves both π-backdonation and σ-donation, which contributes 57.2% and 26.3% to the bonding stabilization of **4**. The π-backbonding contribution in EtC≡CEt and PhC≡CH counterparts is similar to that of **4** (Figure 4). For the dinuclear species, Cu_2_(μ-[3,5-(CF_3_)_2_Pz])_2_(HC≡CH)_2_ (**10**), Cu_2_(μ-[3,5-(CF_3_)_2_Pz])_2_(EtC≡CEt)_2_ (**7**), and Cu_2_(μ-[3,5-(CF_3_)_2_Pz])_2_(HC≡CPh)_2_ (**9**), the Cu-alkyne interaction energy (ΔE_int_) amounts to −43.8, −49.4 and −47.8 kcal⸱mol^−1^, respectively. These values are slightly lower than the somewhat related mononuclear, bis(pyrazolyl)borate species. They, however, involve similar bonding characteristics. The structurally characterized, brominated species Cu_2_(μ-[4-Br-3,5-(CF_3_)_2_Pz])_2_(HC≡CH)_2_ (**2**) with an Cu-alkyne interaction energy of −42.9 kcal⸱mol^−1^, shows a slight destabilization in comparison to Cu_2_(μ-[3,5-(CF_3_)_2_Pz])_2_(HC≡CH)_2_ (**10**). 

We also probed the effect of different substituents on the alkyne moieties by including a phenyl and silyl group –SiMe_3_ (Table 2). In [H_2_B(3,5-(CF_3_)_2_Pz)_2_]Cu(PhC≡CH) (**8**) and [H_2_B(3,5-(CF_3_)_2_Pz)_2_]Cu(HC≡CSiMe_3_) (**5**), the Cu-alkyne stabilization increases slightly due to a small increase of the electrostatic character in comparison to the HC≡CH counterpart (**4**). Moreover, if the silicon atom in the silyl derivative is replaced by a carbon atom, that is—CMe_3_, the stabilization is further improved with an increase in electrostatic contribution. The computational analysis of the Ni(0) complex (Ph_3_P)_2_Ni(HC≡CH)[70] was also performed for a comparison (ESI). As expected, it shows a significantly more stabilized Ni-(HC≡CH) bond (ΔE_int_ = −75.9 kcal⸱mol^−1^) owing to the increase of both electrostatic and orbital stabilizations, as given by a pronounced M→alkyne backbonding, which amounts to −77.6 versus −35.6 kcal⸱mol^−1^ in **4**, which is consistent with the trends of red-shift of the C≡C stretching frequency (or the weakening of the C≡C bond).

Considering the importance of and interest in copper catalyzed azide-alkyne cycloaddition (CuAAC), as well as the rich alkyne chemistry of “[H_2_B(3,5-(CF_3_)_2_Pz)_2_]Cu” moiety, we also set out to explore the use of fluorinated bis(pyrazolyl)borate copper(I) complex [H_2_B(3,5-(CF_3_)_2_Pz)_2_]Cu(NCMe) as a catalyst in cycloaddition of organic azides with terminal alkynes. We also compare the effectiveness of [H_2_B(3,5-(CF_3_)_2_Pz)_2_]Cu(NCMe) with the trinuclear copper(I) pyrazolate {μ-[3,5-(CF_3_)_2_Pz]Cu}_3_ [50], which was found to be quite an effective catalyst for these reactions [47,81]. The Huisgen 1,3-dipolar cycloaddition [82] of organic azides and alkyne received significant attention ever since Sharpless [2] and Meldal [1] independently developed a copper(I) catalyzed reaction. Multidentate nitrogen ligands such as poly(pyrazolyl)borate ligands are known for stabilizing different metal ions including Cu(I) [83,84]. Ruthenium(II) tris(pyrazolyl)borates are reported to catalyze cycloaddition of organic azides and alkyne [85]. There are however very few reports on copper(I) tris(pyrazolyl)borate catalyzed azide-alkyne cycloaddition even though they are known to catalyze several reactions such as cyclopropanation, cyclopropenation, nitrene transfer reactions, etc. [86]. One of the reports concerns the synthesis of N-sulfonyl-1,2,3-triazoles from N-sulfonyl azides and alkynes [87]. That report shows copper(I) supported by tris(pyrazolyl)methanes providing better yields than the tris(pyrazolyl)borate based catalysts. More recent work by Stiriba and co-workers described the use of bis- and tris(pyrazolyl)boratocopper(I) systems to mediate reactions between phenyl- and alkyl-azides with different alkynes to produce 1,4-disubstituted 1,2,3-triazole derivatives moderate to excellent yields [88].

Table 3 summarizes the results of azide-alkyne cycloaddition mediated by the mononuclear [H_2_B(3,5-(CF_3_)_2_Pz)_2_]Cu(NCMe) (**1**) and the trinuclear copper(I) pyrazolate {μ-[3,5-(CF_3_)_2_Pz]Cu}_3_. A reaction of *p*-tolylazide with different terminal alkynes using 1 mol% of catalyst **1** at 110 °C in toluene gives a very high yield for all substrates. The control reaction of *p*-tolyl azide with phenylacetylene without catalyst at the same temperature gives a lower (64%) yield. Thus, the temperature alone drives some of these processes albeit less effectively. We then tested the chemistry using a lower temperature but using a higher catalyst load. The use of 10 mol% compound **1** in EtOH at 40 °C gives triazoles in moderate to excellent yield including a rare CuACC reaction involving the acetylene gas. The control reaction without the catalyst for these conditions gives <1% triazole (reaction of phenylacetylene and *p*-tolylazide). We compared these results to the corresponding {μ-[3,5-(CF_3_)_2_Pz]Cu}_3_ mediated chemistry using 1 mol% catalyst, at room temperature in dichloromethane. This trinuclear catalyst catalyzed alkyne-azide cycloadditions very effectively under milder conditions and generated products in high yield for all substrates except for trimethylsilylacetylene. It is noteworthy that all reactions (except 110 °C reactions) are done in a vial using normal solvents and without using any inert atmosphere. Although we have not probed the mechanistic details, recent work by Larinov and coworkers on {μ-[3,5-(CF_3_)_2_Pz]Cu}_3_ and Stiriba and co-workers on poly(pyrazolyl)borate copper catalyzed cycloadditions suggest different mechanisms, most notably involving tetranuclear and dinuclear intermediates, respectively, for the two systems [88,89]. Better activity of the trinuclear {μ-[3,5-(CF_3_)_2_Pz]Cu}_3_ over mononuclear [H_2_B(3,5-(CF_3_)_2_Pz)_2_]Cu(NCMe) in copper catalyzed azide-alkyne cycloaddition suggests that these reactions involve multicenter catalytic intermediates, consistent with proposed mechanisms [31] and having pre-assembled copper sites with bridging ligands is an advantage [49].

In summary, we report the isolation and characterization of [H_2_B(3,5-(CF_3_)_2_Pz)_2_]Cu(HC≡CH) and [H_2_B(3,5-(CF_3_)_2_Pz)_2_]Cu(HC≡CSiMe_3_) supported by a highly fluorinated bis(pyrazolyl)borate ligand. They feature three-coordinate, trigonal planar copper sites in the solid state and exhibit a significant reduction in ῡ_C__≡C_ value relative to the corresponding free alkyne C≡C stretch. Computational analysis of these molecules and several other related compounds using DFT indicates that the Cu(I)-alkyne interaction in these copper complexes is primarily of electrostatic character. Furthermore, despite the presence of a highly fluorinated ligand in [H_2_B(3,5-(CF_3_)_2_Pz)_2_]Cu(HC≡CH) and [H_2_B(3,5-(CF_3_)_2_Pz)_2_]Cu(HC≡CSiMe_3_), the Cu→alkyne π-backbonding component is much larger than the alkyne→Cu σ-bonding interaction. However, the backbonding is not as high as that computed for (Ph_3_P)_2_Ni(HC≡CH). The mononuclear and dinuclear complexes such as [H_2_B(3,5-(CF_3_)_2_Pz)_2_]Cu(HC≡CH) and Cu_2_(μ-[3,5-(CF_3_)_2_Pz])_2_(HC≡CH)_2_ display similar Cu-alkyne bonding features. The bis(pyrazolyl)borate complex [H_2_B(3,5-(CF_3_)_2_Pz)_2_]Cu(NCMe) catalyzes [3+2] cycloaddition chemistry between tolyl azide and a variety of alkynes including acetylene to produce 1,2,3-triazoles. It is, however, comparatively less effective than the related trinuclear copper catalyst {μ-[3,5-(CF_3_)_2_Pz]Cu}_3_ involving bridging pyrazolates. We are presently exploring the metal mediated alkene and alkyne chemistry supported by these and other fluorinated ligands [90,91].

## 3. Experimental Details

All manipulations except catalysis were carried out under an atmosphere of purified nitrogen using standard Schlenk techniques or in a MBraun glovebox equipped with a −25 °C refrigerator. Solvents were purchased from commercial sources, purified prior to use. NMR spectra were recorded at 25 °C on a JEOL Eclipse 500 spectrometer (Peabody, MA, USA) (^1^H, 500.16 MHz; ^13^C, 125.78 MHz; ^19^F, 470.62 MHz) unless otherwise noted. Proton and carbon chemical shifts are reported in ppm versus Me_4_Si. ^1^H NMR coupling constants (J) are reported in Hertz (Hz) and multiplicities are indicated as follows: s (singlet), d (doublet), t (triplet), m (multiplet). ^19^F NMR values were referenced to external CFCl_3_. Melting points were obtained on a Mel-Temp II apparatus (Wayne, PA, USA) and were not corrected. Elemental analyses were performed using a Perkin–Elmer Model 2400 CHN analyzer (Waltham, MA, USA). IR spectra were collected at room temperature on a Shimadzu IR Prestige-21 FTIR (Kyoto, Japan) containing an ATR attachment using pure liquid or solid materials, with instrument resolution at 2 cm^−1^. Raman data were collected on a Horiba Jobin Yvon LabRAM Aramin Raman spectrometer (Edison, NJ, USA) with a HeNe laser source of 633 nm, by placing pure liquid or solid materials on a glass slide/cuvette. Heating was accomplished by either a heating mantle or a silicone oil bath. Purification of reaction products was carried out by flash column chromatography using silica gel 60 (230–400 mesh). TLC visualization was accompanied by UV light or KMnO_4_ stains. The [H_2_B(3,5-(CF_3_)_2_Pz)_2_]Cu(NCMe) (**1**)[42] and {μ-[3,5-(CF_3_)_2_Pz]Cu}_3_ [50] were prepared according to reported literature procedures. *p*-Tolyl azide was prepared according to the literature procedure [92]. All other reactants and reagents were purchased from commercial sources. Acetylene gas was freed from acetone and purified before use [61]. All other reactants and reagents were purchased from commercial sources.

**Warning**. Due care must be taken when working with acetylene gas. It is known to produce explosive combinations with oxygen, and also form potentially explosive acetylides and other materials with copper salts [62].

*[H_2_B(3,5-(CF_3_)_2_Pz)_2_]Cu(HC≡CH) (**4**):* [H_2_B(3,5-(CF_3_)_2_Pz)_2_]Cu(NCMe) (0.15 g, 0.29 mmol) was dissolved in 7 mL dichloromethane and stirred for ~10 min while bubbling acetylene. The reaction mixture was concentrated with continuous flow of acetylene and kept at −20 °C to obtain X-ray quality colorless crystals of [H_2_B(3,5-(CF_3_)_2_Pz)_2_]Cu(HC≡CH). Yield: >90%. M.P.: 78–79 °C. Anal. Calc. C_12_H_6_BCuF_12_N_4_: C, 28.34%; H, 1.19%; N, 11.02%. Found: C, 28.18%; H, 1.17%; N, 11.04%. ^1^H NMR (CDCl_3_): δ (ppm) 6.93 (s, 2H, Pz*H*), 4.70 (s, 2H, ≡C*H*), 4.00 (br, 2H, B*H*). ^19^F NMR (CDCl_3_): δ (ppm) −59.8 (s), −60.7 (s). ^13^C{^1^H} NMR (CDCl_3_): δ (ppm) 142.2 (q, ^2^*J*_C-F_ = 39.6 Hz, C-3/C-5), 139.9 (q, ^2^*J*_C-F_ = 41.2 Hz, C-3/C-5), 120.1 (q, ^1^*J*_C-F_ =268.7 Hz, CF_3_), 119.2 (q, ^1^*J*_C-F_ =271.1 Hz, CF_3_), 106.4 (C-4), 80.2 (C≡C). ^13^C (^1^H coupled) NMR (CDCl_3_): δ (ppm) 142.2 (q, ^2^*J*_C-F_ = 38.4 Hz, C-3/C-5), 139.9 (q, ^2^*J*_C-F_ = 40.8 Hz, C-3/C-5), 120.1 (q, ^1^*J*_C-F_ =268.7 Hz, CF_3_), 119.2 (q, ^1^*J*_C-F_ =271.1 Hz, CF_3_), 106.4 (d, ^1^*J*_C-H_ = 187.1 Hz, C-4), 80.3 (dd, ^1^*J*_C-H_ = 251.9 Hz, ^2^*J*_C-H_ = 43.2 Hz, C≡C). Raman (cm^−1^), selected peak: 1819 (C≡C).

*[H_2_B(3,5-(CF_3_)_2_Pz)_2_]Cu(HC≡CSiMe_3_) (**5**):* [H_2_B(3,5-(CF_3_)_2_Pz)_2_]Cu(NCMe) (0.15 g, 0.29 mmol) was dissolved in 7 mL dichloromethane. Trimethylsilylacetylene (48 μL, 0.35 mmol) was added to the reaction mixture and stirred overnight. The solvent was evaporated to get white powder product. The product was dissolved in dichloromethane and kept at −20 °C to obtain X-ray quality colorless crystals of [H_2_B(3,5-(CF_3_)_2_Pz)_2_]Cu(HC≡CSiMe_3_). Yield: 91%. M.P.: 59–61 °C. Anal. Calc. C_15_H_14_BCuF_12_N_4_Si: C, 31.02%; H, 2.43%; N, 9.65%. Found: C, 30.64%; H, 2.07%; N, 9.98%. ^1^H NMR (CDCl_3_): δ (ppm) 6.88 (s, 2H, Pz*H*), 4.81 (s, 1H, ≡C*H*), 3.92 (br, 2H, B*H*), 0.24 (s, 9H, Si(C*H*_3_)_3_). ^19^F NMR (CDCl_3_): δ (ppm) −59.9 (s), −61.0 (s). ^13^C{^1^H} NMR (CDCl_3_): δ (ppm) 141.8 (q, ^2^*J*_C-F_ = 38.4 Hz, C-3/C-5), 139.8 (q, ^2^*J*_C-F_ = 43.2 Hz, C-3/C-5), 120.2 (q, ^1^*J*_C-F_ =269.9 Hz, CF_3_), 119.3 (q, ^1^*J*_C-F_ =269.9 Hz, CF_3_), 106.2 (C-4), 97.8 (C≡C), 97.2 (C≡C), −0.2 (Si*C*H_3_). Raman (cm^−1^), selected peak: 1870 (C≡C).

*[H_2_B(3,5-(CF_3_)_2_Pz)_2_]Cu(EtC≡CEt) (**6**):* This was synthesized as reported earlier[6] and crystallized using dichloromethane at −20 °C to obtain crystals suitable for X-ray analysis.

Details of CuACC chemistry involving several alkynes and *p*-tolyl azide

1. General method I for the synthesis of triazoles:

A 50 mL Schlenk flask was charged with the selected alkyne (1.0. mmol), [H_2_B(3,5-(CF_3_)_2_Pz)_2_]Cu(NCMe) (1 mol%) and toluene (5.0 mL) under a nitrogen atmosphere. *p*-tolyl azide (1.0 mmol) was added to the reaction and stirred at 110 °C for 12 h. The solvent was removed under reduced pressure. The residue was dissolved in dichloromethane and filtered through a celite. The dichloromethane was evaporated to get pure product.

2. General method II for the synthesis of triazoles:

A round bottom flask was charged with the selected alkyne (1.0. mmol), p-tolyl azide (1.0 mmol), and EtOH (5.0 mL). [H_2_B(3,5-(CF_3_)_2_Pz)_2_]Cu(NCMe) (10 mol%) was added to the reaction and stirred at 40 °C for 12 h. The solvent was evaporated. The product was isolated using column chromatography (Ethyl acetate/hexanes).

3. General method III for the synthesis of triazoles

A 5 mL vial was charged with selected alkyne (1.0. mmol), p-tolyl azide (1.0 mmol), and dichloromethane (3.0 mL). {μ-[3,5-(CF_3_)_2_Pz]Cu}_3_ (1 mol%) was added to the reaction and stirred at room temperature for 12 h. The yield was calculated using 1,3,5-(trimethoxy)benzene as internal standard.

## 4. X-ray Data Collection and Structure Determinations

A suitable crystal covered with a layer of hydrocarbon/Paratone-N oil was selected and mounted on a Cryo-loop and immediately placed in the low temperature nitrogen stream. The X-ray intensity data were measured at 100(2) K on a Bruker D8 Quest with a Photon 100 CMOS detector equipped with an Oxford Cryosystems (Billerica, MA, USA) 700 series cooler, a graphite monochromator, and a Mo Kα fine-focus sealed tube (λ = 0.71073 Å). Intensity data were processed using the Bruker Apex program suite. Absorption corrections were applied by using SADABS [93]. Initial atomic positions were located by SHELXT [94] and the structures of the compounds were refined by the least-squares method using SHELXL [95] within Olex2 GUI [96]. All the non-hydrogen atoms were refined anisotropically. The hydrogen atoms of BH_2_ moieties as well as acetylenic C≡CH were located in difference Fourier maps, included and refined freely with isotropic displacement parameters. The remaining hydrogen atoms were included in their calculated positions and refined as riding on the atoms to which they are joined. X-ray structural figures were generated using Olex2 [96]. The CCDC 2065196-2065198 files contain the supplementary crystallographic data of [H_2_B(3,5-(CF_3_)_2_Pz)_2_]Cu(HC≡CH) (**4**), [H_2_B(3,5-(CF_3_)_2_Pz)_2_]Cu(HC≡CSiMe_3_) (**5**) and [H_2_B(3,5-(CF_3_)_2_Pz)_2_]Cu(EtC≡CEt) (**6**) [6]. These data can be obtained free of charge via http://www.ccdc.cam.ac.uk/conts/retrieving.html (accessed on 7 December 2021) or from the Cambridge Crystallographic Data Centre (CCDC), 12 Union Road, Cambridge, CB2 1EZ, UK). Additional details are provided in the Supporting Information section.

## 5. Theoretical Methods

Computational details are given as Supporting information.

## Figures and Tables

**Table 1:** Selected bond distances (Å) and angles (º) for [H_2_B(3,5-(CF_3_)_2_Pz)_2_]Cu(HC≡CH) (4), Cu_2_(μ-[4-Br-3,5-(CF_3_)_2_Pz])_2_(HC≡CH)_2_ (2), [Cu{NH(Py)_2_}(HC≡CH)]BF_4_ and [Cu(phen). (HC≡CH)]ClO_4_. Specific bond distances and angles of **4** are presented in CIF and Supporting Information section. Data collection temperatures are in Kelvin. Free acetylene C≡C distance, C≡C stretching frequency, ^1^H and ^13^C chemical shifts are 1.2033(2) Å (gas phase) and 1.193(6) Å (neutron diffraction—less accurate) [71,72], 1974 cm^−1^, 2.01 ppm, and 73.2 ppm [63], respectively.

Complex\ Parameter	4	2	[Cu{NH(Py)_2_} (HC≡CH)]BF_4_	[Cu(phen) (HC≡CH)]ClO_4_
Temp.	100	100	173	283–303
Cu-C	1.972(3) 1.973(3)	1.966(3) 1.974(3)	1.971(3) 1.971(3)	1.930(5) 1.961(5)
C≡C	1.225(5)	1.227(4)	1.188(11)	1.190(7)
Cu-N	1.981(3) 1.981(3)	1.9697(18) 1.9742(18)	1.968(3) 1.968(3)	1.979(4) 1.978(4)
C-Cu-C	36.17(14)	36.29(11)	35.1(3)	35.6(2)
N-Cu-N	96.63(10)	98.94(8)	96.8(2)	84.9(2)
ῡ(C≡C)	1819	1811	1795	1800
^1^H	4.40	4.75	5.21	-
^13^C	80.2	-	-	-
ref	This work	[47]	[57]	[56]

**Table 2:** Energy decomposition analysis of the interaction energy in mononuclear and dinuclear Cu(I) species. Values in kcal·mol^−1^. Vibrational frequencies in cm^−1^.

Mononuclear, Bis(pyrazolyl)Borate Copper Complexes, L = [H_2_B(3,5-(CF_3_)_2_Pz)_2_]; L’ = [H_2_B(4-Br-3,5-(CF_3_)_2_Pz)_2_]										
	LCu(HC≡CH) **4**		LCu(EtC≡CEt) **6**		LCu(PhC≡CH) **8**		LCu(HC≡CSiMe_3_) **5**		L’Cu(HC≡CH)	
ΔE_Pauli_	121.8		131.0		124.8		123.6		119.9	
ΔE_Elstat_	−100.4	59.5%	−112.6	61.7%	−102.3	58.8%	−103.7	59.0%	−99.1	59.5%
ΔE_orb_	−62.2	36.9%	−59.9	32.8%	−61.8	35.5%	−60.3	34.3%	−61.5	36.9%
ΔE_Disp_	−6.0	3.6%	−10.0	5.5%	−10.0	5.7%	−11.7	6.7%	−6.0	3.6%
ΔE_int_	−46.8		−51.5		−49.4		−52.1		−46.7	
π→Cu	−35.6	57.2%	−31.0	51.8%	−33.8	54.6%	−31.5	52.2%	−34.8	56.5%
σ←Cu	−16.3	26.3%	−17.5	29.2%	−16.6	26.9%	−16.7	27.7%	−16.5	26.8%
*v*_C__≡__C__,_ Calc. (Exp.)	1817 (1819)		2056 (2064)		1954 (1927)		1870 (1870)		1818	
**Dinuclear** **, Copper Pyrazolate Complexes**										
	Cu_2_(μ-[3,5-(CF_3_)_2_Pz])_2_(HC≡CH)_2_ **10**		Cu_2_(μ-[3,5-(CF_3_)_2_Pz])_2_(EtC≡CEt)_2_ **7**		Cu_2_(μ-[3,5-(CF_3_)_2_Pz])_2_(PhC≡CH)_2_ **9**			Cu_2_(μ-[4-Br-3,5-(CF_3_)_2_Pz])_2_(HC≡CH)_2_ **2**		
ΔE_Pauli_	128.0		143.2		142.0			124.6		
ΔE_Elstat_	−102.7	59.8%	−118.2	61.3%	−109.8	57.8%		−99.9	59.6%	
ΔE_orb_	−62.8	36.5%	−62.2	32.3%	−65.6	34.5%		−61.5	36.7%	
ΔE_Disp_	−6.3	3.7%	−12.2	6.3%	−14.5	7.6%		−6.1	3.7%	
ΔE_int_	−43.8		−49.4		−47.8			−42.9		
π→Cu	−35.8	57.0%	−32.3	52.0%	−35.7	54.5%		−34.8	56.6%	
σ←Cu	−16.7	26.5%	−17.9	28.7%	−17.0	25.9%		−16.6	27.1%	
*v*_C__≡__C__,_ Calc. (Exp.)	1814		2055 (2033, 2066)		1950			1813 (1811)		

**Table 3:** Azide-alkyne cycloaddition mediated by the mononuclear bis(pyrazolyl)borate [H_2_B(3,5-(CF_3_)_2_Pz)_2_]Cu(NCMe) (**1**) and the trinuclear copper(I) pyrazolate {μ-[3,5-(CF_3_)_2_Pz]Cu}_3_. Different Methods are described in the experimental section.

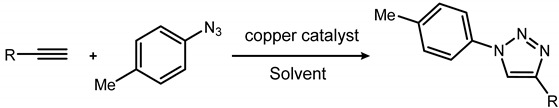				
Entry	Alkyne	Yield (%) [H_2_B(3,5-(CF_3_)_2_Pz)_2_]Cu(NCMe) catalyst		Yield (%) {μ-[3,5-(CF_3_)_2_Pz]Cu}_3_ Catalyst Method III
		Method I	Method II	
1	HC≡CH	-	80	99
2	*n*-PrC≡CH	99	85	99
3	*n*-BuC≡CH	99	89	99
4	*n*-C_8_H_17_C≡CH	99	91	99
5	PhC≡CH	99	84	99
6	Me_3_SiC≡CH	99	56	10

## Supplementary Materials

The following are available online, Table S1. Selected bond distances (Å) and angles (º) and C≡C stretching frequency for mononuclear [H_2_B(3,5-(CF_3_)_2_Pz)_2_]Cu(HC≡CH) (4), [H_2_B(3,5-(CF_3_)_2_Pz)_2_]Cu(EtC≡CEt) (6), and [H_2_B(3,5-(CF_3_)_2_Pz)_2_]Cu(PhC≡CH) (8) (top-row of figures below from L to R), and dinuclear Cu_2_(μ-[4-Br-3,5-(CF_3_)_2_Pz])_2_(HC≡CH)_2_ (2), Cu_2_(μ-[3,5-(CF_3_)_2_Pz])_2_(EtC≡CEt)_2_ (7), and Cu_2_(μ-[3,5-(CF_3_)_2_Pz])_2_(HC≡CPh)_2_ (9) (bottom-row of figures below from L to R), Table S2. Selected NMR spectroscopic (RC≡CR) and C≡C stretching frequency data, Figure S1. ^1^H NMR spectrum of [H_2_B{3,5-(CF_3_)_2_Pz]Cu(HC≡CH) (4) in CDCl_3_, Figure S2: ^13^C{1H} NMR spectrum of [H_2_B{3,5-(CF_3_)_2_Pz]Cu(HC≡CH) (4) in CDCl_3_, Figure S3. ^13^C (^1^H coupled) NMR spectrum of [H_2_B{3,5-(CF_3_)_2_Pz]Cu(HC≡CH) (4) in CDCl_3_, Figure S4. ^19^F NMR spectrum of [H_2_B{3,5-(CF_3_)_2_Pz]Cu(HC≡CH) (4) in CDCl_3_, Figure S5. Raman spectrum of [H_2_B{3,5-(CF_3_)_2_Pz]Cu(HC≡CH) (4), Figure S6. ^1^H NMR spectrum of [H_2_B{3,5-(CF_3_)_2_Pz]Cu(HC≡CSiMe_3_) (5) in CDCl_3_, Figure S7. ^13^C{^1^H} NMR spectrum of [H_2_B{3,5-(CF_3_)_2_Pz]Cu(HC≡CSiMe_3_) (5) in CDCl_3_, Figure S8. ^19^F NMR spectrum of [H_2_B{3,5-(CF_3_)_2_Pz]Cu(HC≡CSiMe_3_) (5) in CDCl_3_, Figure S9. Raman spectrum of [H_2_B{3,5-(CF_3_)_2_Pz]Cu(HC≡CSiMe_3_) (5), Figure S10. ^1^H NMR spectrum of 1-(p-tolyl)-1H-1,2,3-triazole in CDCl_3_, Figure S11. ^13^C{^1^H} NMR spectrum of 1-(p-tolyl)-1H-1,2,3-triazole in CDCl_3_, Figure S12. ^1^H NMR spectrum of 4-propyl-1-(p-tolyl)-1H-1,2,3-triazole in CDCl_3_, Figure S13. ^13^C{^1^H} NMR spectrum of 4-propyl-1-(p-tolyl)-1H-1,2,3-triazole in CDCl_3_, Figure S14. ^1^H NMR spectrum of 4-butyl-1-(p-tolyl)-1H-1,2,3-triazole in CDCl_3_, Figure S15. ^13^C{^1^H} NMR spectrum of 4-butyl-1-(p-tolyl)-1H-1,2,3-triazole in CDCl_3_, Figure S16. ^1^H NMR spectrum of 4-octyl-1-(p-tolyl)-1H-1,2,3-triazole in CDCl_3_, Figure S17. ^13^C{^1^H} NMR spectrum of 4-octyl-1-(p-tolyl)-1H-1,2,3-triazole in CDCl_3_, Figure S18. ^1^H NMR spectrum of 4-phenyl-1-(p-tolyl)-1H-1,2,3-triazole in CDCl_3_, Figure S19. ^13^C{^1^H} NMR spectrum of 4-phenyl-1-(p-tolyl)-1H-1,2,3-triazole in CDCl_3_, Figure S20. ^1^H NMR spectrum of 1-(p-tolyl)-4-(trimethylsilyl)-1H-1,2,3-triazole in CDCl_3_, Figure S21. ^13^C{^1^H} NMR spectrum of 1-(p-tolyl)-4-(trimethylsilyl)-1H-1,2,3-triazole in CDCl_3_, Figure S22. Molecular structure of [H_2_B(3,5-(CF_3_)_2_Pz)_2_]Cu(HC≡CH) (4), Table S3. Crystal data and structure refinement for [H_2_B(3,5-(CF_3_)_2_Pz)_2_]Cu(HC≡CH) (4).Table S4. Bond Lengths for [H_2_B(3,5-(CF_3_)_2_Pz)_2_]Cu(HC≡CH) (4), Table S5. Bond Angles for [H_2_B(3,5-(CF_3_)_2_Pz)_2_]Cu(HC≡CH) (4), Figure S23. Molecular structure of [H_2_B(3,5-(CF_3_)_2_Pz)_2_]Cu(HC≡CSiMe_3_) (5), Table S6. Crystal data and structure refinement for [H_2_B(3,5-(CF_3_)_2_Pz)_2_]Cu(HC≡CSiMe_3_) (5), Table S7. Bond Lengths for [H_2_B(3,5-(CF_3_)_2_Pz)_2_]Cu(HC≡CSiMe_3_) (5), Table S8. Bond Angles for [H_2_B(3,5-(CF_3_)_2_Pz)_2_]Cu(HC≡CSiMe_3_) (5), Figure S24. Molecular structure of [H_2_B(3,5-(CF_3_)_2_Pz)_2_]Cu(EtC≡CEt)(6), Table S9. Crystal data and structure refinement for [H_2_B(3,5-(CF_3_)_2_Pz)_2_]Cu(EtC≡CEt)(6), Table S10. Bond Lengths for [H_2_B(3,5-(CF_3_)_2_Pz)_2_]Cu(EtC≡CEt)(6), Table S11. Bond Angles for [H_2_B(3,5-(CF_3_)_2_Pz)_2_]Cu(EtC≡CEt)(6), Figure S25. Raman spectrum of Trimethylsilylacetylene, Figure S26. Raman spectrum of 3-hexyne, Figure S27. Raman spectrum of Phenylacetylene, Table S12. Energy decomposition analysis of the interaction energy for (Ph_3_P)_2_Ni(HC≡CH) species accounting for the acetylene coordination. Values in kcal·mol^−1^. Vibrational frequencies in cm^−1^. Experimental value from [23], Figure S28. Deformation densities account for the π-backbonding (left) and σ-donation (right) contribution to the bonding scheme in the formation of (Ph_3_P)_2_Ni(HC≡CH). Charge flow from red to blue.

## Sample Availability

Samples of the compounds are not available from authors. Synthetic details are in the experimental section.
