# Supplementary material for: Terminal and Internal Alkyne Complexes and Azide-Alkyne Cycloaddition Chemistry of Copper(I) Supported by a Fluorinated Bis(pyrazolyl)borate
Source: Molecules. 2021 Dec 21;27(1):16. doi: 10.3390/molecules27010016 (PMC8746352; doi:10.3390/molecules27010016)
Supplement: Supplementary file 1 [file molecules-27-00016-s001.zip › molecules-1521967-supplementary.pdf]

Terminal and internal alkyne complexes and azide-alkyne cycloaddition chemistry of copper(I) supported by a fluorinated bis(pyrazolyl)borate

Anurag Noonikara-Poyil,[1] Alvaro Muñoz-Castro, [2] and H. V. Rasika Dias[1]

Affiliations:

[1] Department of Chemistry and Biochemistry, The University of Texas at Arlington, Arlington, Texas 76019, United States, Email: dias@uta.edu

[2] Grupo de Química Inorgánica y Materiales Moleculares, Facultad de Ingenieria, Universidad Autonoma de Chile, El Llano Subercaseaux 2801, Santiago, Chile, Email: alvaro.munoz@uautonoma.cl

Table of Contents

Selected bond distances, angles, and C≡C stretching frequency S-2
Spectroscopic data comparison of metal complexes S-3
^{1}H, ^{13}C, ^{19}F NMR Spectra and Raman spectra of Metal complexes............. S-4
Characterization of Triazoles.. S-9
^{1}H and ^{13}C NMR Spectra of Triazoles... S-11
X-ray data and structure determinations.. S-17
Raman spectra of free Alkyne S-29
Computational details S-31
References... S-33

Table S1. Selected bond distances (Å) and angles (º) and C≡C stretching frequency for mononuclear $[H_2B(3,5\text{-}(CF_3)_2Pz)_2]Cu(HC{\equiv}CH)$ (**4**), $[H_2B(3,5\text{-}(CF_3)_2Pz)_2]Cu(EtC{\equiv}CEt)$ (**6**), and $[H_2B(3,5\text{-}(CF_3)_2Pz)_2]Cu(PhC{\equiv}CH)$ (**8**) (top-row of figures below from L to R), and dinuclear $Cu_2(\mu\text{-}[4\text{-}Br\text{-}3,5\text{-}(CF_3)_2Pz])_2(HC{\equiv}CH)_2$ (**2**), $Cu_2(\mu\text{-}[3,5\text{-}(CF_3)_2Pz])_2(EtC{\equiv}CEt)_2$ (**7**), and $Cu_2(\mu\text{-}[3,5\text{-}(CF_3)_2Pz])_2(HC{\equiv}CPh)_2$ (**9**) (bottom-row of figures below from L to R).

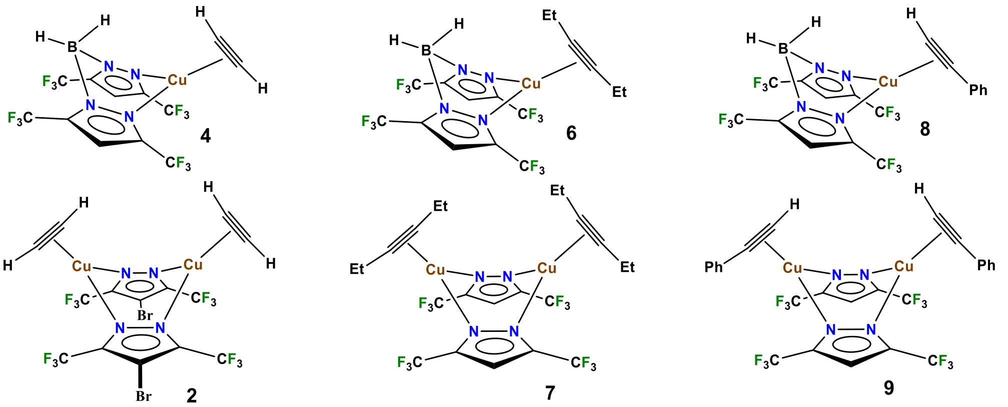

Parameter\ Complex	$[H_2B(3,5\text{-}(CF_3)_2Pz)_2]Cu(HC{\equiv}CH)$ (**4**)	$Cu_2(\mu\text{-}[4\text{-}Br\text{-}3,5\text{-}(CF_3)_2Pz])_2(HC{\equiv}CH)_2$ (**2**)*	$[H_2B(3,5\text{-}(CF_3)_2Pz)_2]Cu(EtC{\equiv}CEt)$ (**6**)	$Cu_2(\mu\text{-}[3,5\text{-}(CF_3)_2Pz])_2(EtC{\equiv}CEt)_2$ (**7**)	$[H_2B(3,5\text{-}(CF_3)_2Pz)_2]Cu(HC{\equiv}CPh)$ (**8**)	$Cu_2(\mu\text{-}[3,5\text{-}(CF_3)_2Pz])_2(HC{\equiv}CPh)_2$ (**9**)**
Cu-C	1.972(3)<br>1.973(3)	1.966(3)<br>1.974(3)	1.9818(16)<br>1.9862(16)	1.9844(13)<br>1.9766(17)<br>1.9741(18)<br>1.9758(16)	1.936(4)<br>2.003(4)	1.944(3)<br>1.987(3)<br>1.944(3)<br>2.014(3)
C≡C	1.225(5)	1.227(4)	1.235(2)	1.225(3)<br>1.228(3)	1.205(6)	1.228(4)<br>1.225(4)
Cu-N	1.981(3)<br>1.981(3)	1.9697(18)<br>1.9742(18)	1.9992(14)<br>2.0022(14)	1.9750(14)<br>1.9921(14)<br>1.9873(14)<br>1.9850(14)	1.989(3)<br>1.991(3)	1.976(2)<br>1.962(2)<br>1.986(2)<br>1.967(2)<br>1.969(2)<br>1.984(2)<br>1.958(2)<br>1.968(2)
C-Cu-C	36.17(14)	36.29(11)	36.26(7)	36.03(8)<br>36.22(7)	35.56(18)	36.38(12)<br>36.01(13)<br>35.91(12)<br>36.66(12)
N-Cu-N	96.63(10)	98.94(8)	91.94(6)	96.62(6)<br>98.35(6)	94.16(11)	99.77(9)<br>100.57(9)<br>100.98(9)<br>98.88(9)
C≡C-C	-	-	160.33(17)<br>162.90(17)	161.72(18)<br>161.5(2)<br>160.30(18)<br>161.19(19)	163.5(4)	160.7(3)<br>162.2(3)<br>162.4(3)<br>160.0(3)
$\bar{\upsilon}$(C≡C)	1819	1811	2064	2033, 2066	1927	1918, 1928, 1950
ref	*This work*	[1]	This work and [2]	*[3]*	*[4]*	*[1]*

*$Cu_2(\mu\text{-}[4\text{-}Br\text{-}3,5\text{-}(CF_3)_2Pz])_2(HC{\equiv}CH)_2$ sites on a plane of symmetry

**There are two molecules of $Cu_2(\mu\text{-}[3,5\text{-}(CF_3)_2Pz])_2(HC{\equiv}CPh)_2$ in the asymmetric unit

Table S2: Selected NMR spectroscopic (RC≡CR) and C≡C stretching frequency data.

Compound	Raman/IR (cm^{-1})	^{1}H NMR (ppm, inCDCl$_3$) (≡C*H* peak)	^{13}C{^{1}H} NMR (ppm, in CDCl$_3$) (C≡C peaks)	Reference
[$H_2B(3,5-(CF_3)_2Pz)_2$]Cu(HC≡CH) (**4**)	1819	4.70	80.2	This paper
Free Acetylene	1974	1.90	73.2	[5]
Cu_2(μ-[4-Br-3,5-$(CF_3)_2$Pz])$_2$(HC≡CH)$_2$ (**2**)	1811	4.75 (- 70 °C)	-	[1]
[Cu-(NH(py)$_2$)(C_2H_2)]BF_4	1795	5.21/5.59*	-	[6,7]
[Cu(phen)(HC≡CH)]ClO_4	1800	-	-	[8]
Cu_4(μ-[3,5-$(CF_3)_2$Pz])$_4$(μ-HC≡CH)$_2$ (**3**)	1638	6.16	79.2	[1]
[$H_2B(3,5-(CF_3)_2Pz)_2$]Cu(HC≡CSiMe$_3$) (**5**)	1870	4.81	97.2, 97.8	This paper
Trimethylsilylacetylene	2107	2.36	90.2, 93.1	-
[$H_2B(3,5-(CF_3)_2Pz)_2$]Cu(PhC≡CH) (**8**)	1927	4.60	79.2, 95.4#	[4]
Phenylacetylene	2111	3.21	78.8, 84.2	-
Cu_2(μ-[3,5-$(CF_3)_2$Pz])$_2$(HC≡CPh)$_2$ (**9**)	1918, 1928, 1950	3.17	77.8, 84.5	[1]
[$H_2B(3,5-(CF_3)_2Pz)_2$]Cu(EtC≡CEt) (**6**)	2064	-	91.0	This paper and [2]
3-Hexyne	2231, 2247, 2298	-	81.0	-
Cu_2(μ-[3,5-$(CF_3)_2$Pz])$_2$(EtC≡CEt)$_2$ (**7**)	2033, 2066	-	82.5	[3]

*NMR spectrum in $(CD_3)_2CO$, #NMR spectrum in C_6D_6

^{1}H, ^{13}C, and ^{19}F NMR Spectra of Metal Complexes

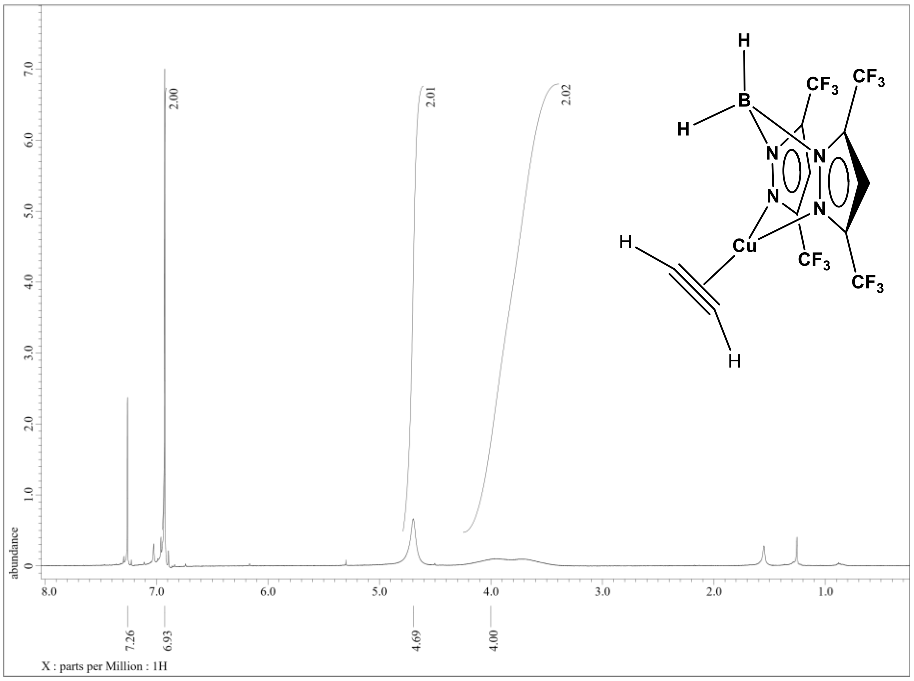

Figure S1: ^{1}H NMR spectrum of $[H_2B(3,5\text{-}(CF_3)_2Pz)_2]Cu(HC{\equiv}CH)$ (**4**) in $CDCl_3$.

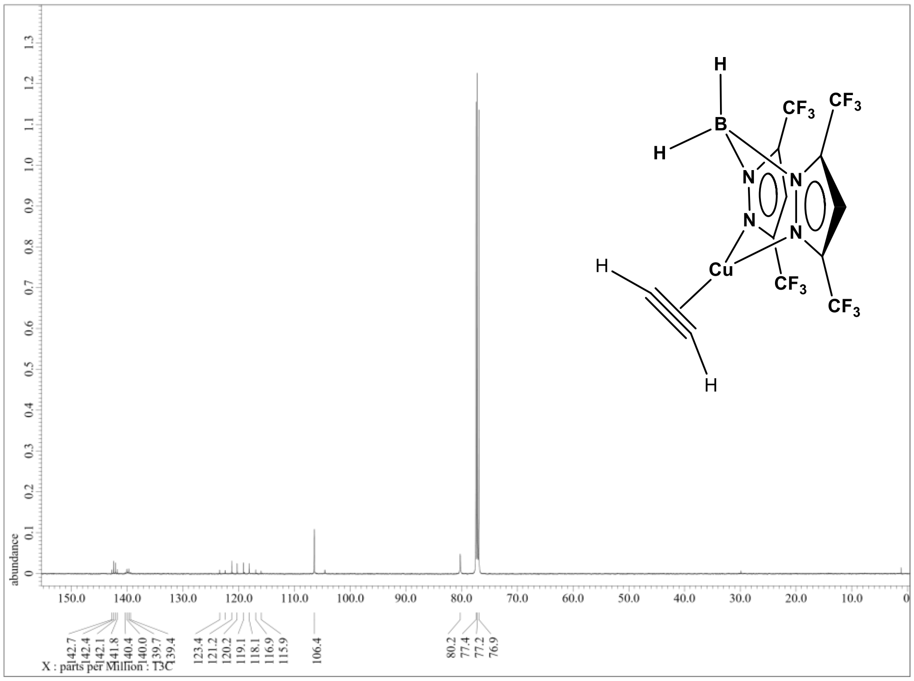

Figure S2: $^{13}C\{^{1}H\}$ NMR spectrum of $[H_2B(3,5\text{-}(CF_3)_2Pz)_2]Cu(HC{\equiv}CH)$ (**4**) in $CDCl_3$.

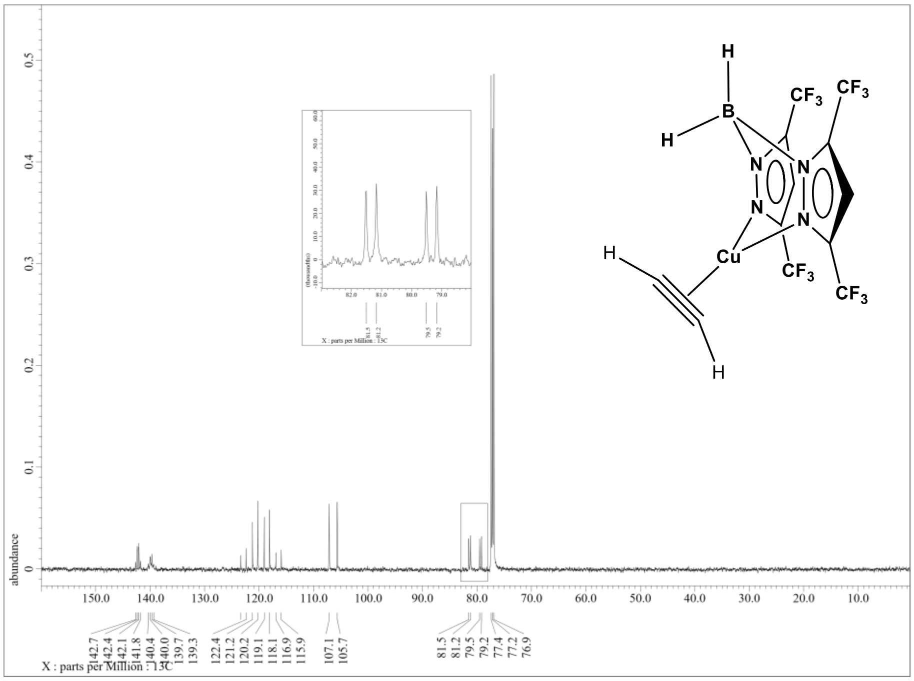

Figure S3: ^{13}C (^{1}H coupled) NMR spectrum of [$H_2B(3,5\text{-}(CF_3)_2Pz)_2$]Cu(HC≡CH) (**4**) in $CDCl_3$.

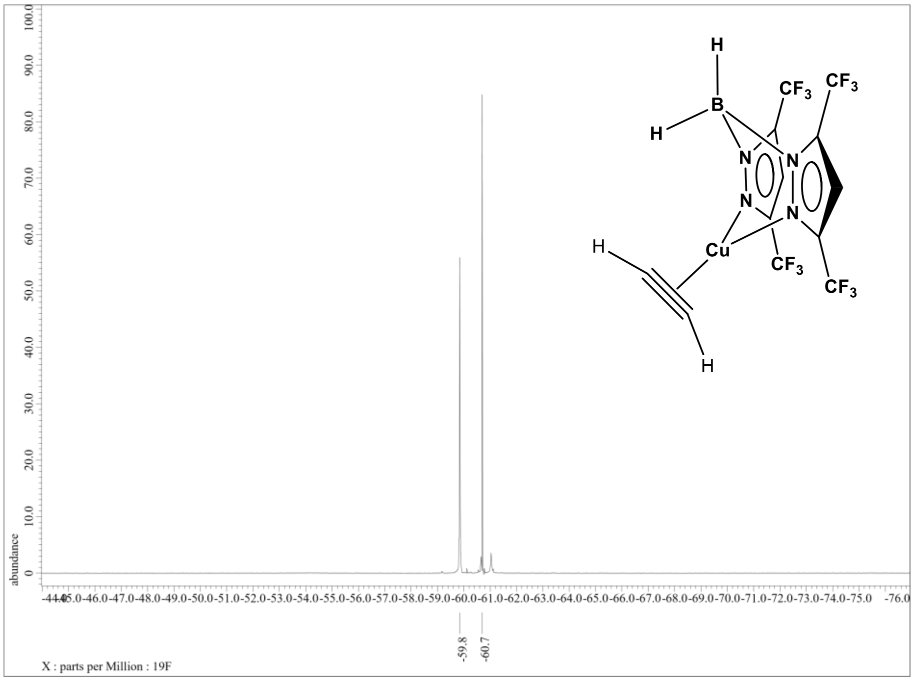

Figure S4: ^{19}F NMR spectrum of [$H_2B(3,5\text{-}(CF_3)_2Pz)_2$]Cu(HC≡CH) (**4**) in $CDCl_3$.

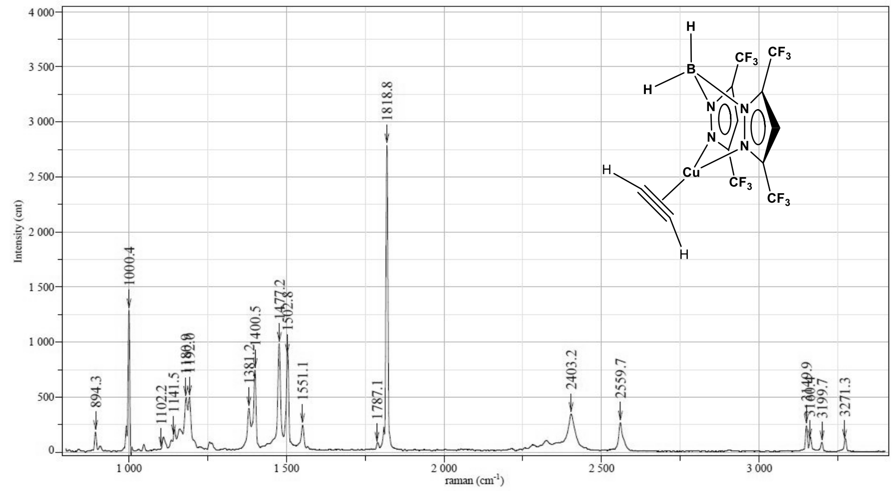

Exposition	5	Slit	100
Accumulation	5 x 4	Operator	
Laser	632.817	Sample	
Spectro	Multi	Remark	
Hole	200	Power	

Figure S5: Raman spectrum of $[H_2B(3,5\text{-}(CF_3)_2Pz)_2]Cu(HC{\equiv}CH)$ (**4**).

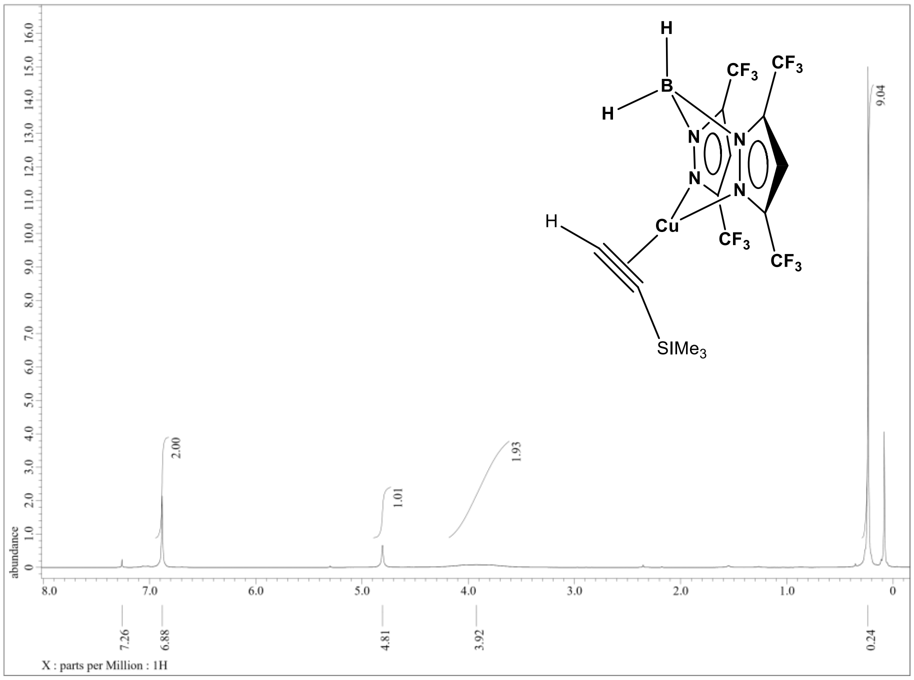

Figure S6: ^{1}H NMR spectrum of $[H_2B(3,5\text{-}(CF_3)_2Pz)_2]Cu(HC{\equiv}CSiMe_3)$ (**5**) in $CDCl_3$.

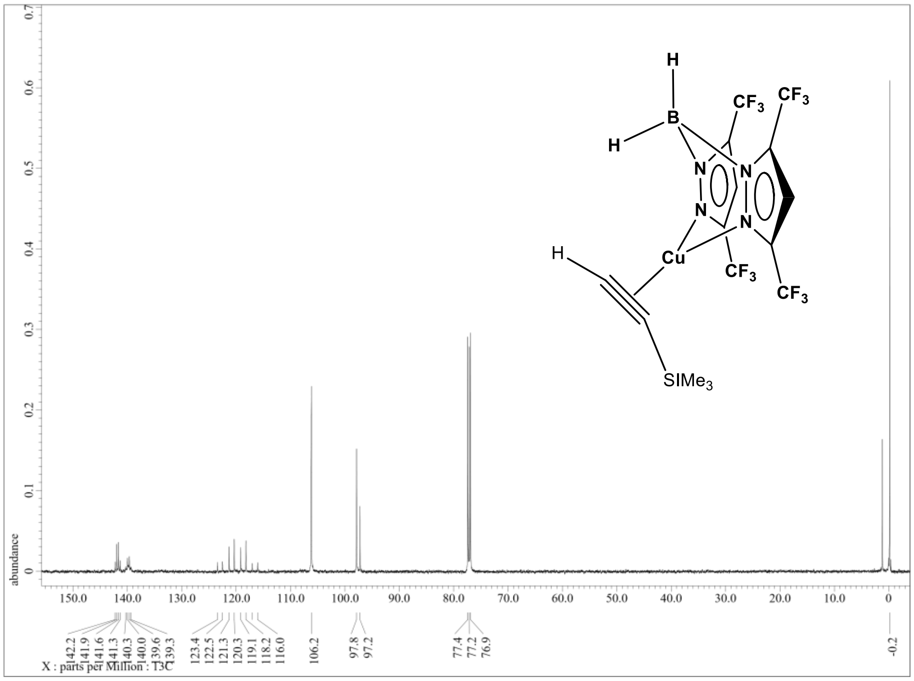

Figure S7: $^{13}C\{^{1}H\}$ NMR spectrum of $[H_2B(3,5\text{-}(CF_3)_2Pz)_2]Cu(HC{\equiv}CSiMe_3)$ (**5**) in $CDCl_3$.

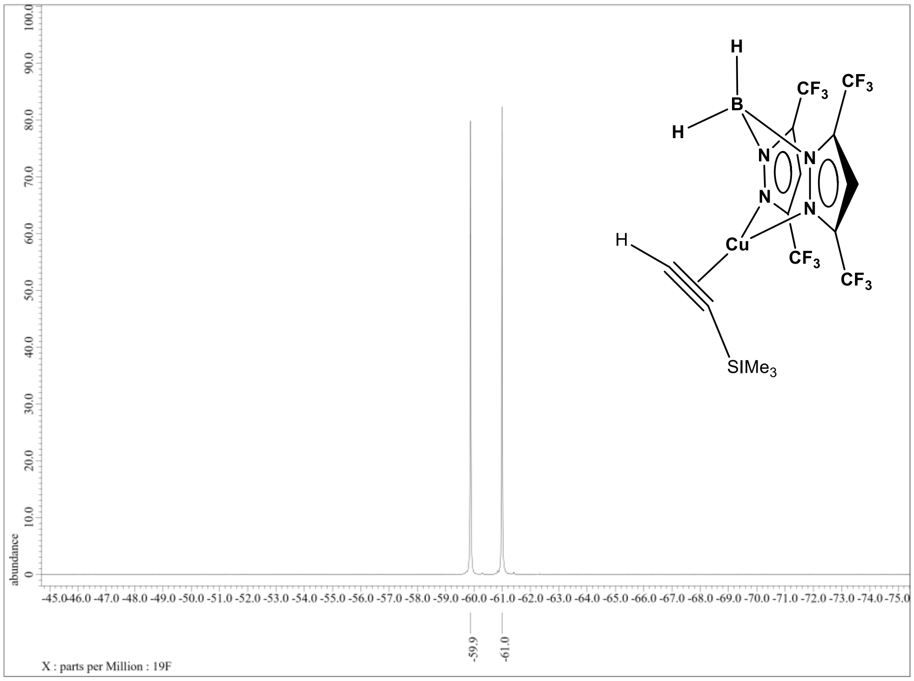

Figure S8: ^{19}F NMR spectrum of $[H_2B(3,5\text{-}(CF_3)_2Pz)_2]Cu(HC{\equiv}CSiMe_3)$ (**5**) in $CDCl_3$.

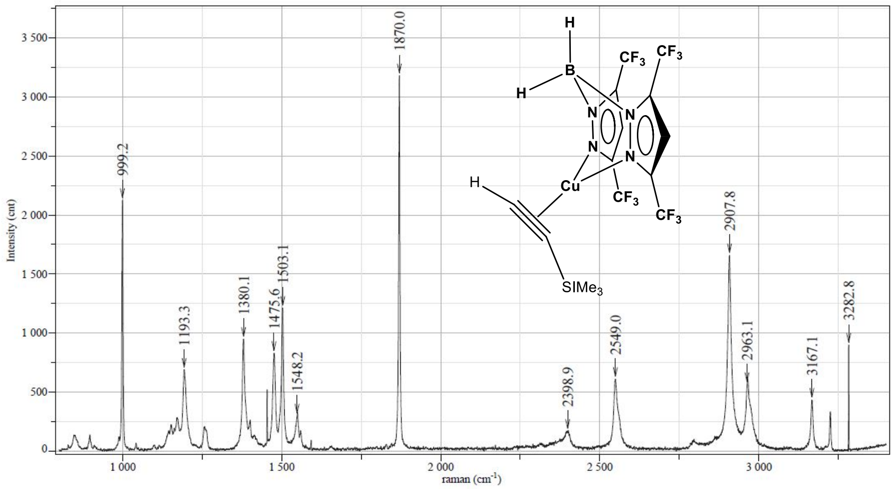

Exposition	5	Slit	100
Accumulation	1 x 21	Operator	Anurag
Laser	632.817	Sample	3,5-CF3BpCu_TMSacetylen
Spectro	Auto	Remark	
Hole	200	Power	

Figure S9: Raman spectrum of $[H_2B(3,5-(CF_3)_2Pz)_2]Cu(HC{\equiv}CSiMe_3)$ (**5**).

Spectral data of Triazoles

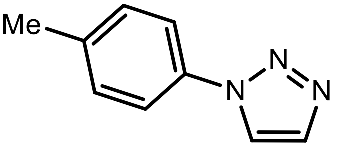

1-(*p*-tolyl)-1*H*-1,2,3-triazole[1]: From acetylene and *p*-tolyl azide as described in the general method (see above). ^{1}H NMR (500 MHz, $CDCl_3$): δ 8.01 (s, 1H, =CH), 7.86 (s, 1H, =CH), 7.60 (d, *J* = 8.0 Hz, 2H, Ar*H*), 7.30 (d, *J* = 8.0 Hz, 2H, Ar*H*), 2.40 (s, 3H, ArCH_3). ^{13}C{^{1}H} NMR (125 MHz, $CDCl_3$): δ 139.1, 134.8 (2C), 130.4, 122.3, 120.7, 21.2.

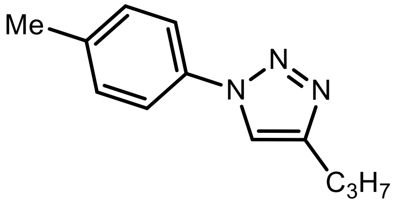

4-propyl-1-(*p*-tolyl)-1*H*-1,2,3-triazole[9]: From 1-Pentyne and *p*-tolyl azide as described in the general method (see above). ^{1}H NMR (500 MHz, $CDCl_3$): δ 7.68 (s, 1H, =CH), 7.59 (d, *J* = 8.0 Hz, 2H, Ar*H*), 7.29 (d, *J* = 8.0 Hz, 2H, Ar*H*), 2.76 (t, *J* = 7.5 Hz, 2H, =CCH_2), 2.41 (s, 3H, ArCH_3), 1.78-1.73 (m, 2H, =CCH_2CH_2), 1.01 (t, *J* = 7.5 Hz, 3H, =CCH_2CH_2CH_3). ^{13}C{^{1}H} NMR (125 MHz, $CDCl_3$): δ 149.0, 138.5, 135.1, 130.2, 120.4, 119.0, 27.8, 22.8, 21.2, 13.9.

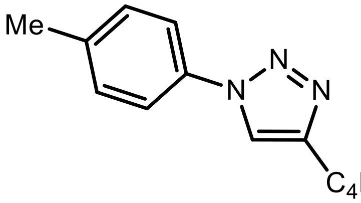

4-butyl-1-(*p*-tolyl)-1*H*-1,2,3-triazole[9]: From 1-Hexyne and *p*-tolyl azide as described in the general method (see above). ^{1}H NMR (500 MHz, $CDCl_3$): δ 7.67 (s, 1H, =CH), 7.50 (d, *J* = 6.9 Hz, 2H, Ar*H*), 7.17 (d, *J* = 6.9 Hz, 2H, Ar*H*), 2.70 (t, *J* = 6.9 Hz, 2H, =CCH_2), 2.29 (s, 3H, ArCH_3), 1.64-1.61 (m, 2H, =CCH_2CH_2), 1.36-1.32 (m, 2H, =CCH_2CH_2CH_2), 0.87 (t, *J* = 6.9 Hz, 3H, =C$CH_2CH_2CH_2$CH_3). ^{13}C{^{1}H} NMR (125 MHz, $CDCl_3$): δ 148.9, 138.2, 134.8, 129.9, 120.0, 118.8, 31.4, 25.2, 22.2, 20.8, 13.7.

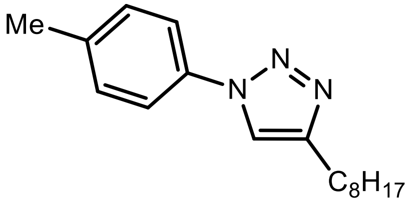

4-octyl-1-(*p*-tolyl)-1*H*-1,2,3-triazole[10]: From 1-Decyne and *p*-tolyl azide as described in the general method (see above). ^{1}H NMR (500 MHz, $CDCl_3$): δ 7.68 (s, 1H, =CH), 7.59 (d, *J* = 8.0 Hz, 2H, Ar*H*), 7.29 (d, *J* = 8.0 Hz, 2H, Ar*H*), 2.78 (t, *J* = 7.5 Hz, 2H, =CCH_2), 2.41 (s, 3H, ArCH_3), 1.75-1.69 (m, 2H, =CCH_2CH_2), 1.41-1.27 (m, 10H, =CCH_2CH_2CH_2CH_2CH_2CH_2CH_2), 0.88 (t, *J* = 7.5 Hz, 3H, =C$CH_2CH_2CH_2CH_2CH_2CH_2CH_2CH_3$). ^{13}C NMR{^{1}H} (125 MHz, $CDCl_3$): δ 149.2, 138.6, 135.2, 130.3, 120.5, 118.9, 32.0, 29.6, 29.5, 29.4, 29.3, 25.9, 22.8, 21.2, 14.3.

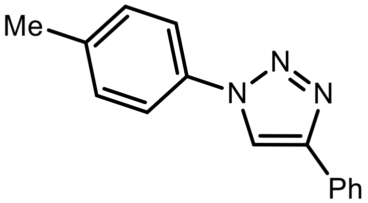

4-phenyl-1-(*p*-tolyl)-1*H*-1,2,3-triazole[9]: From Phenylacetylene and *p*-tolyl azide as described in the general method (see above). ^{1}H NMR (500 MHz, $CDCl_3$): δ 8.16 (s, 1H, =CH), 7.92-7.90 (m, 2H, Ar*H*), 7.67-7.65 (m, 2H, Ar*H*), 7.47-7.44 (m, 2H, Ar*H*), 7.38-7.32 (m, 3H, Ar*H*), 2.43 (s, 3H, ArCH_3). ^{13}C{^{1}H} NMR (125 MHz, $CDCl_3$): δ 148.4, 139.0, 134.9, 130.5, 130.4, 129.0, 128.5, 125.9, 120.5, 117.7, 21.2.

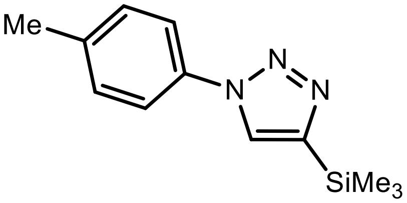

1-(*p*-tolyl)-4-(trimethylsilyl)-1*H*-1,2,3-triazole[11]: From Trimethylsilylacetylene and *p*-tolyl azide as described in the general method (see above). ^{1}H NMR (500 MHz, $CDCl_3$): δ 7.93 (s, 1H, =CH), 7.53 (d, *J* = 8.6 Hz, 2H, Ar*H*), 7.19 (d, *J* = 8.6 Hz, 2H, Ar*H*), 2.31 (s, 3H, ArCH_3), 0.31 (s, 9H, Si(CH_3)$_3$). ^{13}C{^{1}H} NMR (125 MHz, $CDCl_3$): δ 147.0, 138.3, 134.6, 130.0, 127.2, 120.5, 20.9, -1.2.

^{1}H and ^{13}C NMR Spectra of triazoles

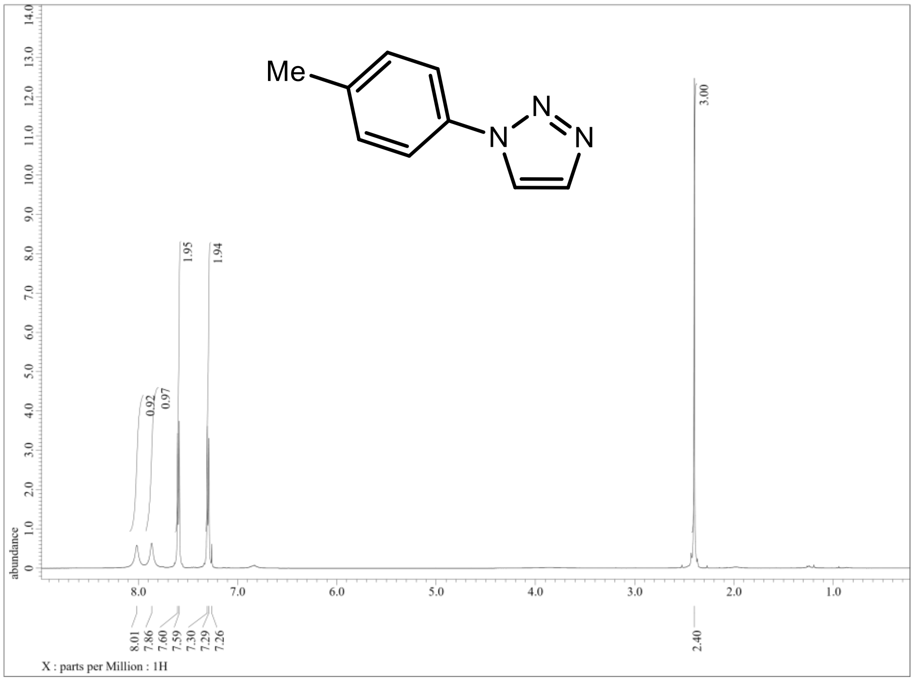

Figure S10: ^{1}H NMR spectrum of **1-(*p*-tolyl)-1*H*-1,2,3-triazole** in $CDCl_3$.

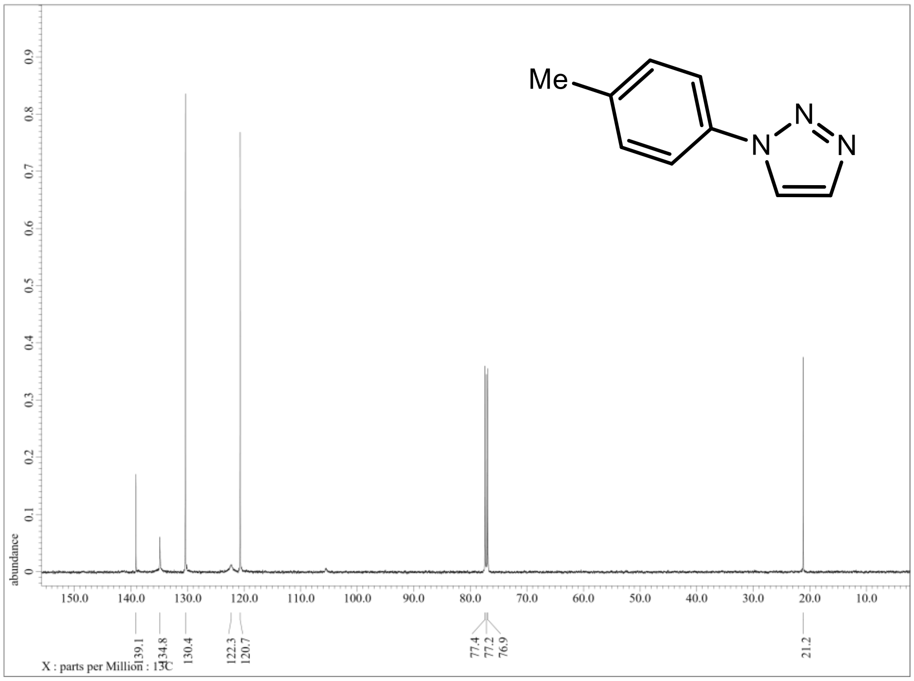

Figure S11: $^{13}C\{^{1}H\}$ NMR spectrum of **1-(*p*-tolyl)-1*H*-1,2,3-triazole** in $CDCl_3$.

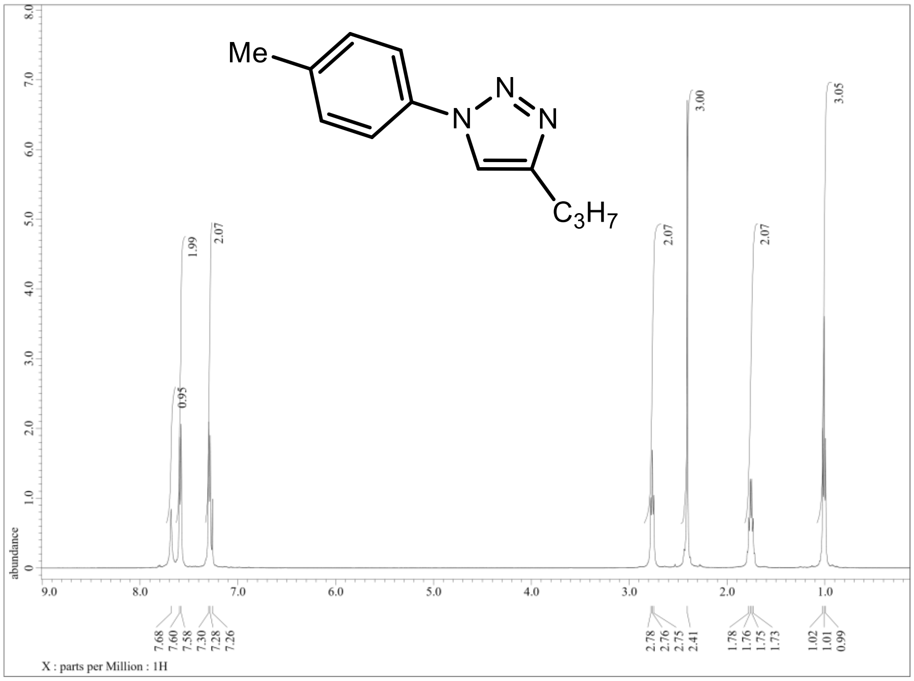

Figure S12: ^{1}H NMR spectrum of **4-propyl-1-(*p*-tolyl)-1*H*-1,2,3-triazole** in $CDCl_3$.

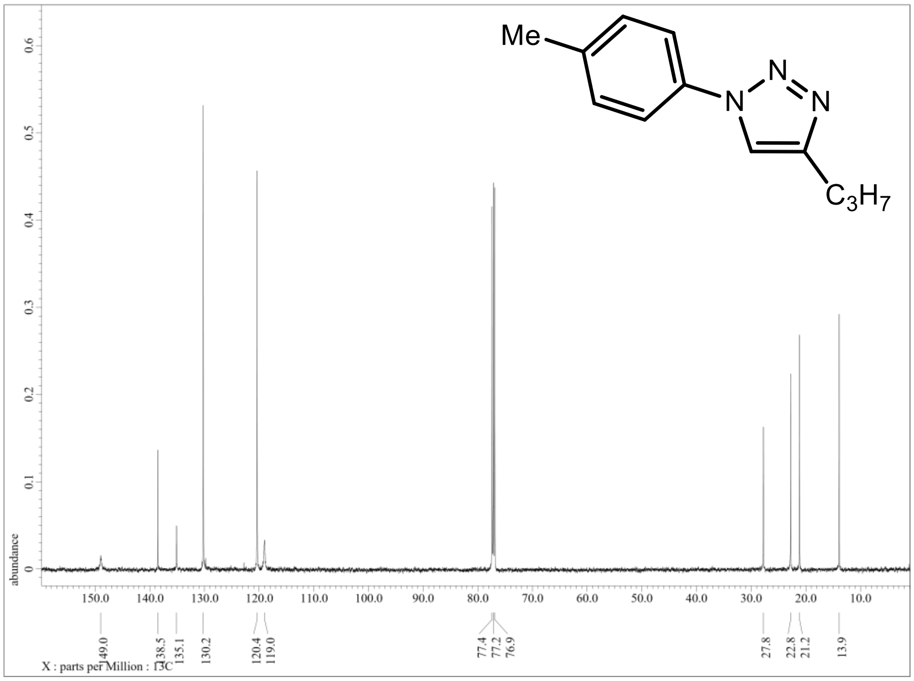

Figure S13: $^{13}C\{^{1}H\}$ NMR spectrum of **4-propyl-1-(*p*-tolyl)-1*H*-1,2,3-triazole** in $CDCl_3$.

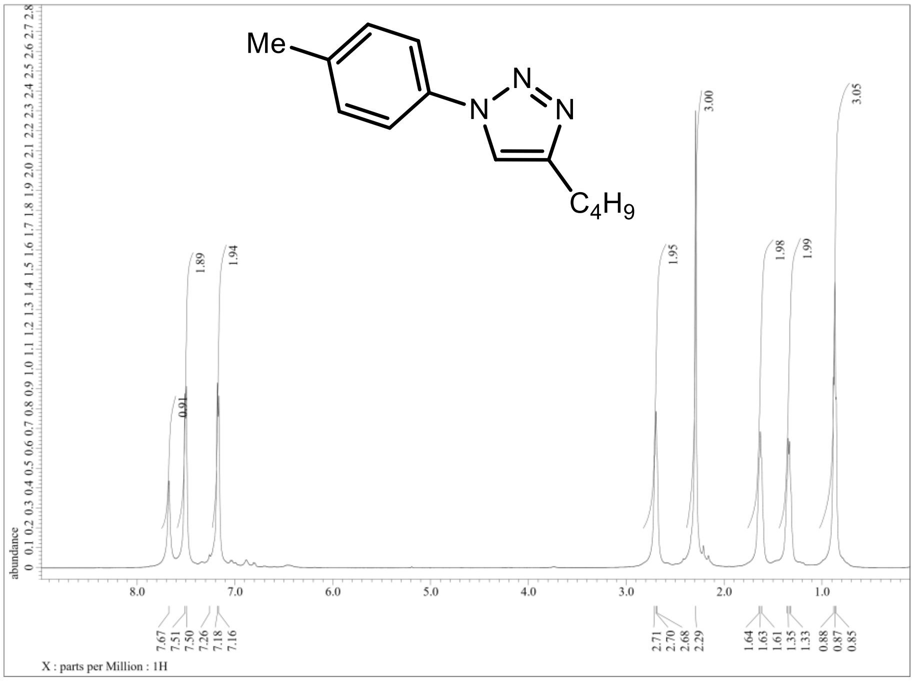

Figure S14: ^{1}H NMR spectrum of **4-butyl-1-(*p*-tolyl)-1*H*-1,2,3-triazole** in $CDCl_3$.

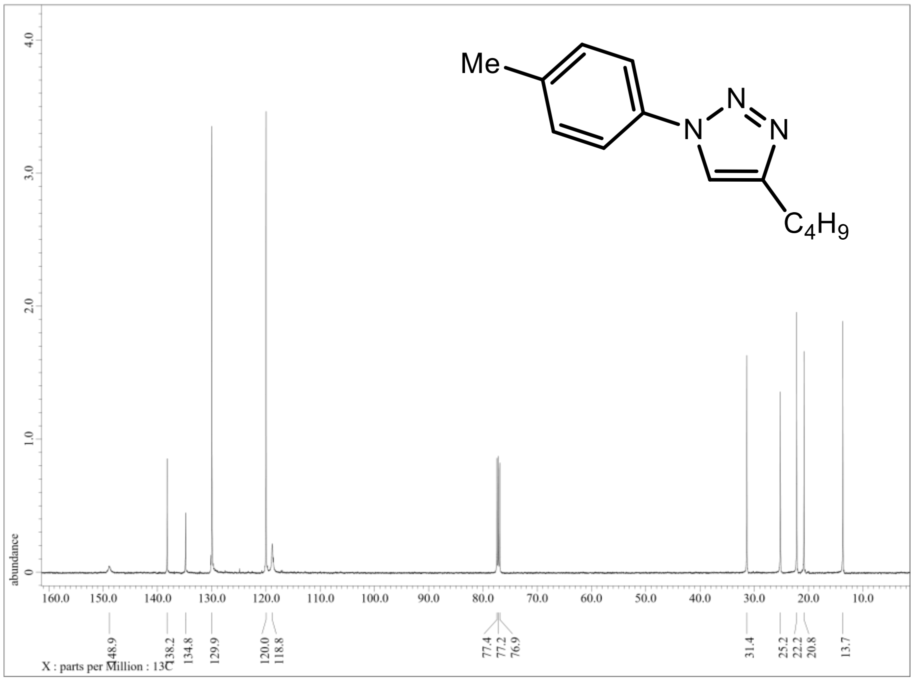

Figure S15: $^{13}C\{^{1}H\}$ NMR spectrum of **4-butyl-1-(*p*-tolyl)-1*H*-1,2,3-triazole** in $CDCl_3$.

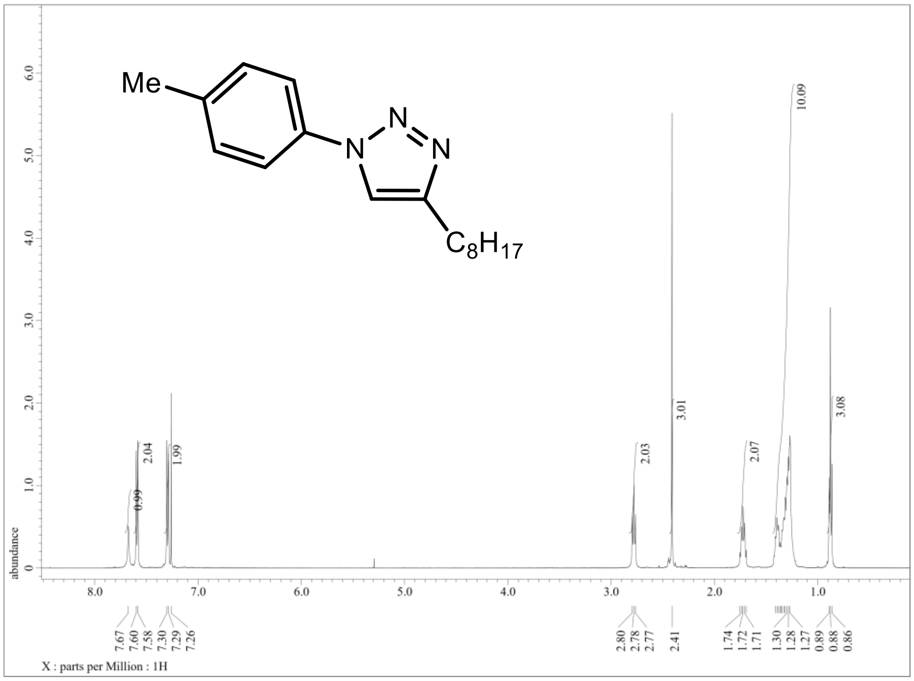

Figure S16: ^{1}H NMR spectrum of **4-octyl-1-(*p*-tolyl)-1*H*-1,2,3-triazole** in $CDCl_3$.

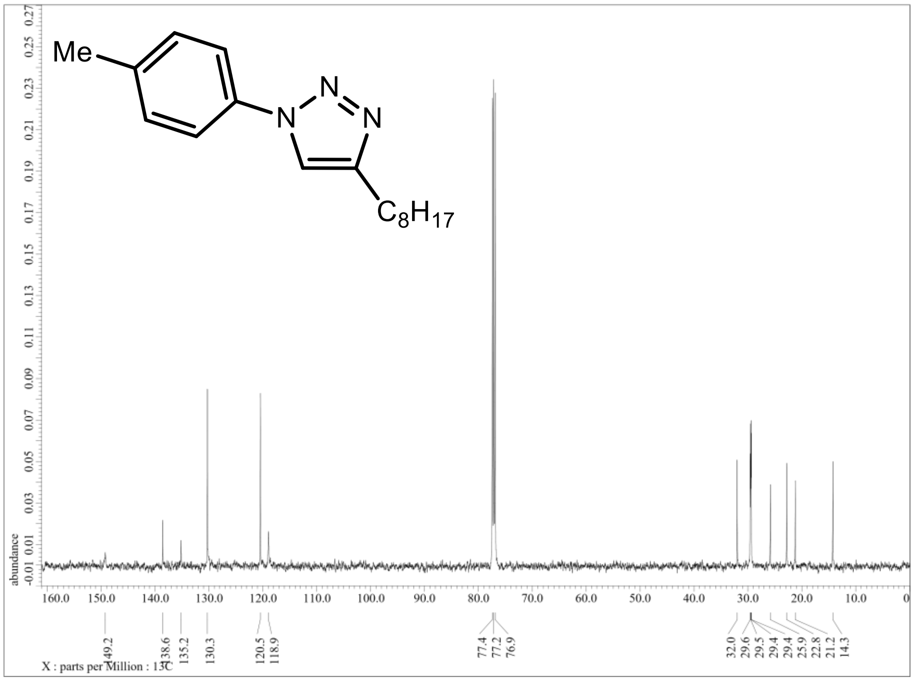

Figure S17: $^{13}C\{^{1}H\}$ NMR spectrum of **4-octyl-1-(*p*-tolyl)-1*H*-1,2,3-triazole** in $CDCl_3$.

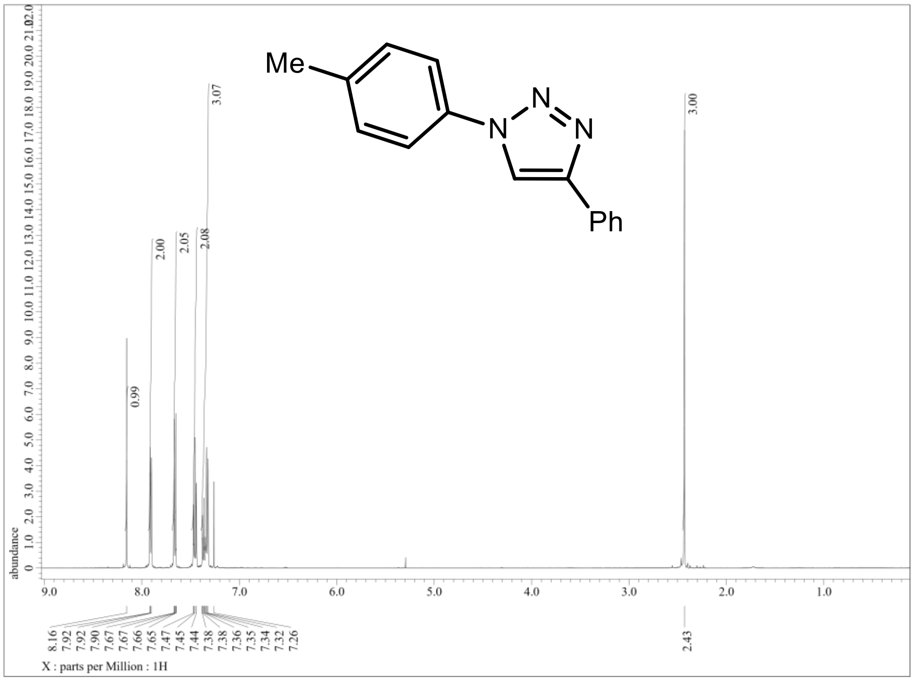

Figure S18: ^{1}H NMR spectrum of **4-phenyl-1-(*p*-tolyl)-1*H*-1,2,3-triazole** in $CDCl_3$.

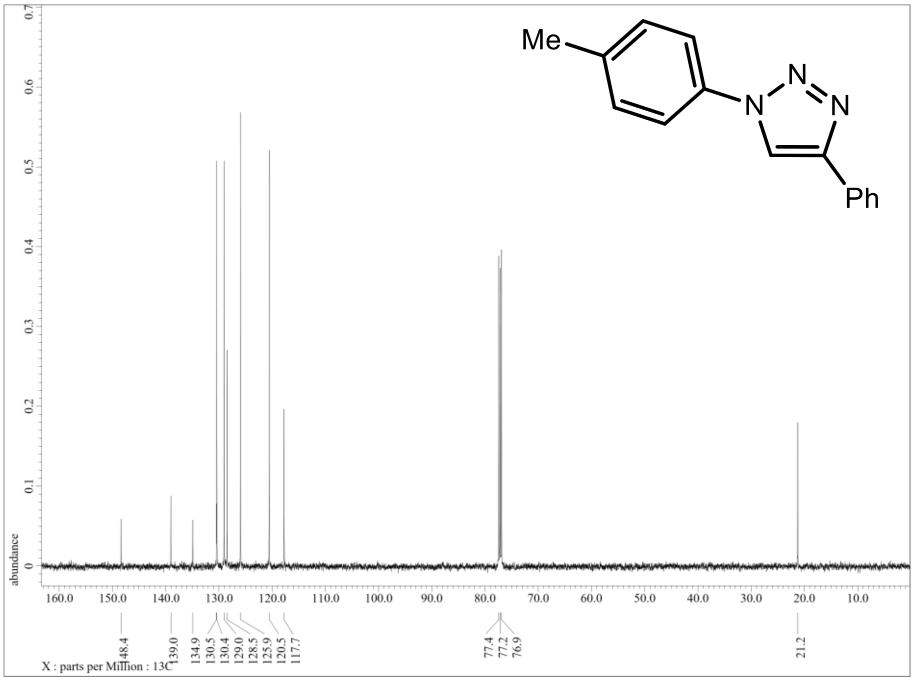

Figure S19: ^{13}C{^{1}H} NMR spectrum of **4-phenyl-1-(*p*-tolyl)-1*H*-1,2,3-triazole** in $CDCl_3$.

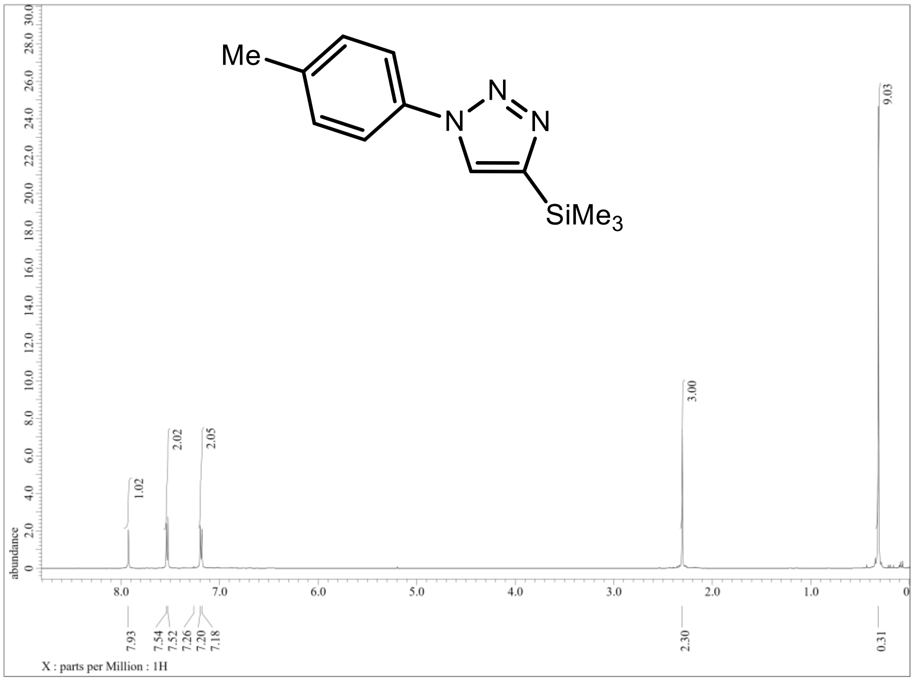

Figure S20: ^{1}H NMR spectrum of **1-(*p*-tolyl)-4-(trimethylsilyl)-1*H*-1,2,3-triazole** in $CDCl_3$.

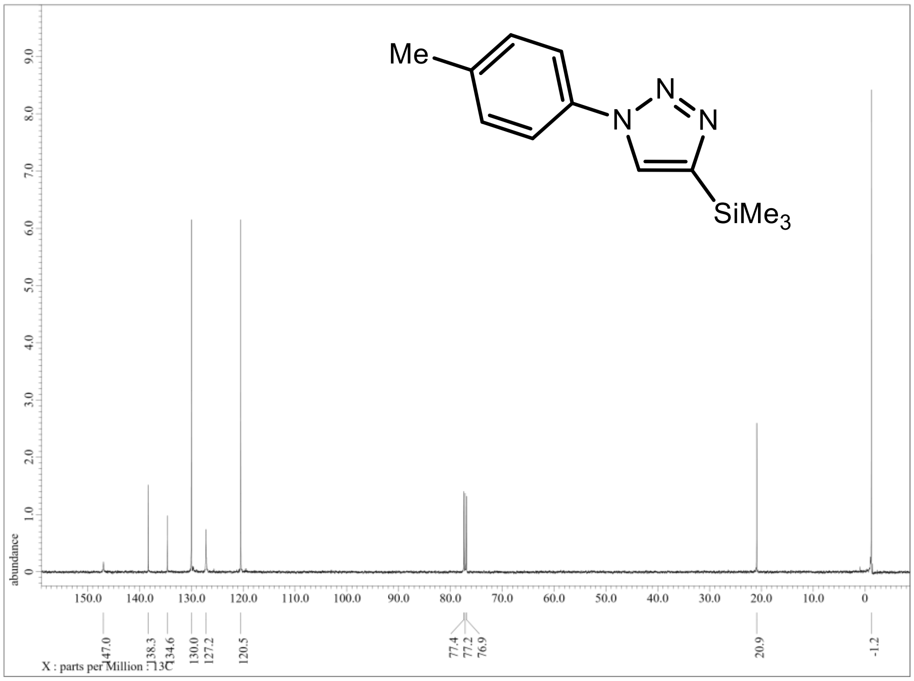

Figure S21: $^{13}C\{^{1}H\}$ NMR spectrum of **1-(*p*-tolyl)-4-(trimethylsilyl)-1*H*-1,2,3-triazole** in $CDCl_3$.

X-ray data and structure determinations

[$H_2B(3,5-(CF_3)_2Pz)_2$]Cu(HC≡CH)

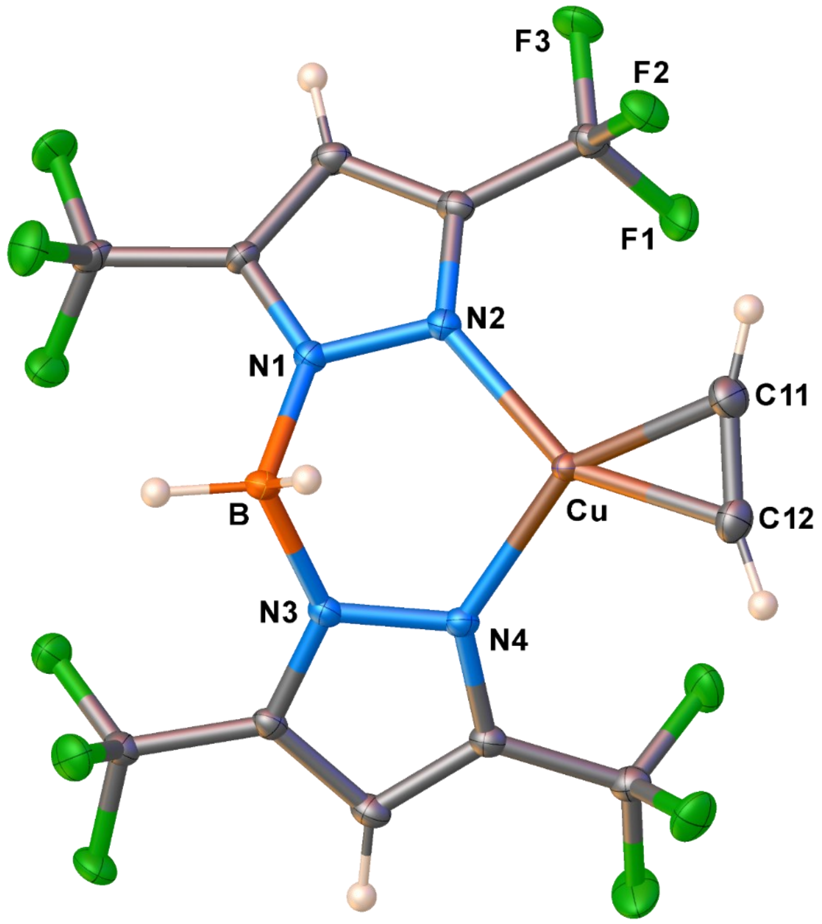

Figure S22: Molecular structure of [$H_2B(3,5-(CF_3)_2Pz)_2$]Cu(HC≡CH) (**4**).

Table S3: Crystal data and structure refinement for [$H_2B(3,5-(CF_3)_2Pz)_2$]Cu(HC≡CH) (**4**).

Identification code	rad685_0m_a
Empirical formula	$C_{12}H_6BCuF_{12}N_4$
Formula weight	508.56
Temperature/K	99.98
Crystal system	monoclinic
Space group	$P2_1/n$
a/Å	9.2406(5)
b/Å	9.3114(5)

c/Å	19.4623(10)
α/°	90
β/°	90.690(2)
γ/°	90
Volume/Å^3	1674.47(15)
Z	4
ρ_{calc}g/cm^3	2.017
μ/mm^{-1}	1.439
F(000)	992.0
Crystal size/mm^3	0.27 × 0.13 × 0.035
Radiation	Mo Kα (λ = 0.71073)
2Θ range for data collection/°	6.044 to 61.108
Index ranges	-13 ≤ h ≤ 13, -13 ≤ k ≤ 13, -27 ≤ l ≤ 27
Reflections collected	22428
Independent reflections	5110 [R_{int} = 0.0358, R_{sigma} = 0.0304]
Data/restraints/parameters	5110/0/289
Goodness-of-fit on F^2	1.106
Final R indexes [I>=2σ (I)]	R_1 = 0.0435, wR_2 = 0.1093
Final R indexes [all data]	R_1 = 0.0519, wR_2 = 0.1182
Largest diff. peak/hole / e Å^{-3}	0.76/-0.56

Table S4: Bond Lengths for [$H_2B(3,5-(CF_3)_2Pz)_2$]Cu(HC≡CH) (**4**).

**Atom**	**Atom**	**Length/Å**	**Atom**	**Atom**	**Length/Å**
Cu	N2	1.981(3)	N1	C3	1.350(4)
Cu	N4	1.981(3)	N1	B	1.570(4)
Cu	C11	1.972(3)	N2	C1	1.333(4)
Cu	C12	1.973(3)	N3	N4	1.357(4)
F1	C4	1.341(4)	N3	C8	1.350(4)
F2	C4	1.343(4)	N3	B	1.569(4)
F3	C4	1.341(4)	N4	C6	1.336(4)
F4	C5	1.334(4)	C1	C2	1.388(4)
F5	C5	1.340(4)	C1	C4	1.487(4)
F6	C5	1.334(3)	C2	C3	1.383(4)
F7	C9	1.338(4)	C3	C5	1.496(4)
F8	C9	1.339(4)	C6	C7	1.388(4)
F9	C9	1.328(4)	C6	C9	1.497(4)
F10	C10	1.342(3)	C7	C8	1.391(4)

Table S4: Bond Lengths for [$H_2B(3,5-(CF_3)_2Pz)_2$]Cu(HC≡CH) (**4**).

Atom	Atom	Length/Å	Atom	Atom	Length/Å
F11	C10	1.338(4)	C8	C10	1.492(4)
F12	C10	1.331(4)	C11	C12	1.225(5)
N1	N2	1.360(4)			

Table S5: Bond Angles for [$H_2B(3,5-(CF_3)_2Pz)_2$]Cu(HC≡CH) (**4**).

Atom	Atom	Atom	Angle/°	Atom	Atom	Atom	Angle/°
N4	Cu	N2	96.63(10)	F3	C4	C1	110.4(3)
C11	Cu	N2	114.01(13)	F4	C5	F5	106.7(3)
C11	Cu	N4	149.36(13)	F4	C5	F6	107.4(3)
C11	Cu	C12	36.17(14)	F4	C5	C3	112.6(3)
C12	Cu	N2	150.15(13)	F5	C5	C3	112.1(3)
C12	Cu	N4	113.19(13)	F6	C5	F5	107.4(3)
N2	N1	B	118.8(2)	F6	C5	C3	110.3(3)
C3	N1	N2	108.7(2)	N4	C6	C7	111.7(3)
C3	N1	B	132.2(3)	N4	C6	C9	120.1(3)
N1	N2	Cu	116.36(19)	C7	C6	C9	128.1(3)
C1	N2	Cu	136.2(2)	C6	C7	C8	103.1(3)
C1	N2	N1	106.6(2)	N3	C8	C7	109.6(3)
N4	N3	B	118.9(2)	N3	C8	C10	122.7(3)
C8	N3	N4	108.8(2)	C7	C8	C10	127.6(3)
C8	N3	B	132.1(3)	F7	C9	F8	106.8(3)
N3	N4	Cu	117.10(18)	F7	C9	C6	110.8(3)
C6	N4	Cu	136.1(2)	F8	C9	C6	112.3(3)
C6	N4	N3	106.7(2)	F9	C9	F7	107.8(3)
N2	C1	C2	111.7(3)	F9	C9	F8	106.4(3)
N2	C1	C4	120.4(3)	F9	C9	C6	112.4(3)
C2	C1	C4	127.9(3)	F10	C10	C8	109.6(3)
C3	C2	C1	103.3(3)	F11	C10	F10	107.2(2)
N1	C3	C2	109.7(3)	F11	C10	C8	111.6(3)
N1	C3	C5	123.6(3)	F12	C10	F10	107.3(3)
C2	C3	C5	126.7(3)	F12	C10	F11	107.5(3)
F1	C4	F2	106.3(3)	F12	C10	C8	113.4(3)
F1	C4	F3	107.5(3)	C12	C11	Cu	72.0(2)
F1	C4	C1	113.0(3)	C11	C12	Cu	71.9(2)

Table S5: Bond Angles for $[H_2B(3,5\text{-}(CF_3)_2Pz)_2]Cu(HC{\equiv}CH)$ (**4**).

**Atom**	**Atom**	**Atom**	**Angle/°**	**Atom**	**Atom**	**Atom**	**Angle/°**
F2	C4	C1	112.3(3)	N3	B	N1	107.6(2)
F3	C4	F2	107.0(3)				

Crystal Data for $C_{12}H_6BCuF_{12}N_4$ (M =508.56 g/mol): monoclinic, space group $P2_1/n$ (no. 14), a = 9.2406(5) Å, b = 9.3114(5) Å, c = 19.4623(10) Å, β = 90.690(2)°, V = 1674.47(15) Å^3, Z = 4, T = 99.98 K, μ(Mo Kα) = 1.439 mm^{-1}, $Dcalc$ = 2.017 g/cm^3, 22428 reflections measured (6.044° ≤ 2Θ ≤ 61.108°), 5110 unique (R_{int} = 0.0358, R_{sigma} = 0.0304) which were used in all calculations. The final R_1 was 0.0435 (I > 2σ(I)) and wR_2 was 0.1182 (all data).

[$H_2B(3,5-(CF_3)_2Pz)_2]Cu(HC{\equiv}CSiMe_3)$

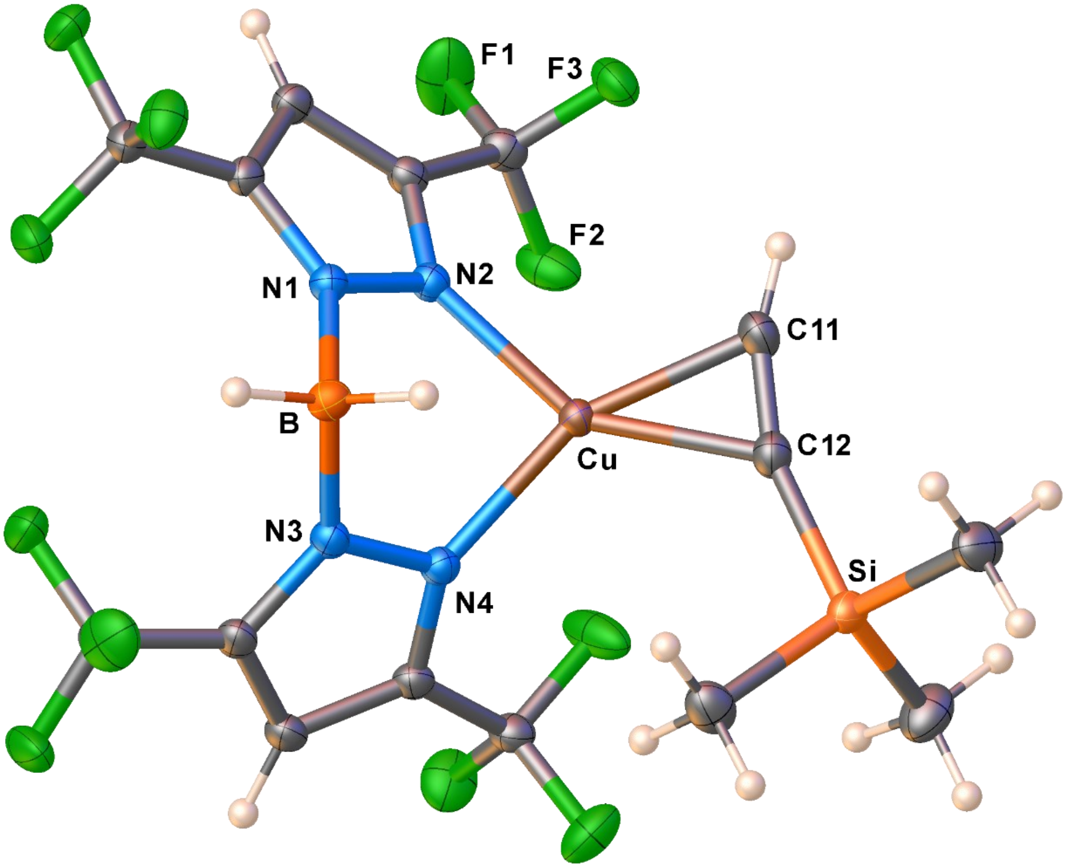

Figure S23:Molecular structure of $[H_2B(3,5-(CF_3)_2Pz)_2]Cu(HC{\equiv}CSiMe_3)$ (**5**).

Table S6: Crystal data and structure refinement for $[H_2B(3,5-(CF_3)_2Pz)_2]Cu(HC{\equiv}CSiMe_3)$ (**5**).

Identification code	rad773_0m_a
Empirical formula	$C_{15}H_{14}BCuF_{12}N_4Si$
Formula weight	580.74
Temperature/K	99.99
Crystal system	monoclinic
Space group	$P2_1/c$
a/Å	12.8899(4)
b/Å	13.6339(4)
c/Å	12.9411(4)
α/°	90

β/°	97.8970(10)
γ/°	90
Volume/Å^3	2252.70(12)
Z	4
ρ_{calc}g/cm^3	1.712
μ/mm^{-1}	1.132
F(000)	1152.0
Crystal size/mm^3	0.33 × 0.23 × 0.2
Radiation	Mo Kα (λ = 0.71073)
2Θ range for data collection/°	5.656 to 75.966
Index ranges	-22 ≤ h ≤ 21, -23 ≤ k ≤ 23, -21 ≤ l ≤ 22
Reflections collected	45484
Independent reflections	11851 [R_{int} = 0.0343, R_{sigma} = 0.0354]
Data/restraints/parameters	11851/0/323
Goodness-of-fit on F^2	1.021
Final R indexes [I>=2σ (I)]	R_1 = 0.0372, wR_2 = 0.0821
Final R indexes [all data]	R_1 = 0.0576, wR_2 = 0.0902
Largest diff. peak/hole / e Å^{-3}	0.58/-0.48

Table S7: Bond Lengths for $[H_2B(3,5\text{-}(CF_3)_2Pz)_2]Cu(HC{\equiv}CSiMe_3)$ (**5**).

Atom	Atom	Length/Å	Atom	Atom	Length/Å
Cu	N2	1.9857(9)	F12	C10	1.3344(15)
Cu	N4	1.9845(9)	N1	N2	1.3513(13)
Cu	C11	1.9600(12)	N1	C3	1.3501(14)
Cu	C12	1.9957(11)	N1	B	1.5778(15)
Si	C12	1.8713(12)	N2	C1	1.3344(14)
Si	C13	1.8572(14)	N3	N4	1.3521(13)
Si	C14	1.8551(15)	N3	C8	1.3530(14)
Si	C15	1.8579(13)	N3	B	1.5737(16)
F1	C4	1.3311(14)	N4	C6	1.3355(14)
F2	C4	1.3382(15)	C1	C2	1.3914(16)
F3	C4	1.3359(14)	C1	C4	1.4875(16)
F4	C5	1.3363(14)	C2	C3	1.3842(16)
F5	C5	1.3392(14)	C3	C5	1.4928(16)
F6	C5	1.3327(13)	C6	C7	1.3923(16)
F7	C9	1.3388(14)	C6	C9	1.4893(16)
F8	C9	1.3272(14)	C7	C8	1.3799(17)
F9	C9	1.3344(14)	C8	C10	1.4945(16)

Table S7: Bond Lengths for $[H_2B(3,5-(CF_3)_2Pz)_2]Cu(HC\equiv CSiMe_3)$ (**5**).

**Atom**	**Atom**	**Length/Å**	**Atom**	**Atom**	**Length/Å**
F10	C10	1.3319(15)	C11	C12	1.2343(17)
F11	C10	1.3389(17)			

Table S8: Bond Angles for $[H_2B(3,5-(CF_3)_2Pz)_2]Cu(HC\equiv CSiMe_3)$ (**5**).

**Atom**	**Atom**	**Atom**	**Angle/°**	**Atom**	**Atom**	**Atom**	**Angle/°**
N2	Cu	C12	148.36(4)	F2	C4	C1	111.56(9)
N4	Cu	N2	90.59(4)	F3	C4	F2	106.16(10)
N4	Cu	C12	120.05(4)	F3	C4	C1	112.60(10)
C11	Cu	N2	112.92(5)	F4	C5	F5	107.31(10)
C11	Cu	N4	156.37(5)	F4	C5	C3	109.78(10)
C11	Cu	C12	36.35(5)	F5	C5	C3	111.82(9)
C13	Si	C12	108.64(6)	F6	C5	F4	107.33(9)
C13	Si	C15	111.59(7)	F6	C5	F5	107.17(10)
C14	Si	C12	107.92(7)	F6	C5	C3	113.16(10)
C14	Si	C13	112.45(7)	N4	C6	C7	111.06(10)
C14	Si	C15	111.59(7)	N4	C6	C9	120.11(10)
C15	Si	C12	104.21(6)	C7	C6	C9	128.76(10)
N2	N1	B	117.79(9)	C8	C7	C6	103.61(10)
C3	N1	N2	108.71(9)	N3	C8	C7	109.51(10)
C3	N1	B	133.49(9)	N3	C8	C10	122.98(11)
N1	N2	Cu	115.16(7)	C7	C8	C10	127.47(11)
C1	N2	Cu	137.31(8)	F7	C9	C6	111.42(9)
C1	N2	N1	107.11(9)	F8	C9	F7	107.08(10)
N4	N3	C8	108.77(9)	F8	C9	F9	106.89(10)
N4	N3	B	117.34(9)	F8	C9	C6	111.70(10)
C8	N3	B	133.68(10)	F9	C9	F7	106.18(10)
N3	N4	Cu	115.68(7)	F9	C9	C6	113.18(10)
C6	N4	Cu	135.95(8)	F10	C10	F11	106.87(11)
C6	N4	N3	107.03(9)	F10	C10	F12	107.64(11)
N2	C1	C2	111.18(10)	F10	C10	C8	112.37(10)
N2	C1	C4	119.19(10)	F11	C10	C8	112.38(11)
C2	C1	C4	129.62(10)	F12	C10	F11	107.41(10)
C3	C2	C1	103.34(9)	F12	C10	C8	109.93(11)
N1	C3	C2	109.64(10)	C12	C11	Cu	73.40(8)

Table S8: Bond Angles for $[H_2B(3,5\text{-}(CF_3)_2Pz)_2]Cu(HC{\equiv}CSiMe_3)$ (**5**).

Atom	Atom	Atom	Angle/°	Atom	Atom	Atom	Angle/°
N1	C3	C5	123.19(10)	Si	C12	Cu	128.26(6)
C2	C3	C5	127.11(10)	C11	C12	Cu	70.25(8)
F1	C4	F2	107.99(11)	C11	C12	Si	160.64(11)
F1	C4	F3	107.00(10)	N3	B	N1	106.41(9)
F1	C4	C1	111.23(10)				

Crystal Data for $C_{15}H_{14}BCuF_{12}N_4Si$ (M =580.74 g/mol): monoclinic, space group $P2_1/c$ (no. 14), a = 12.8899(4) Å, b = 13.6339(4) Å, c = 12.9411(4) Å, β = 97.8970(10)°, V = 2252.70(12) Å^3, Z = 4, T = 99.99 K, μ(Mo Kα) = 1.132 mm^{-1}, $Dcalc$ = 1.712 g/cm^3, 45484 reflections measured (5.656° ≤ 2Θ ≤ 75.966°), 11851 unique (R_{int} = 0.0343, R_{sigma} = 0.0354) which were used in all calculations. The final R_1 was 0.0372 (I > 2σ(I)) and wR_2 was 0.0902 (all data).

[$H_2B(3,5\text{-}(CF_3)_2Pz)_2$]Cu(EtC≡CEt)

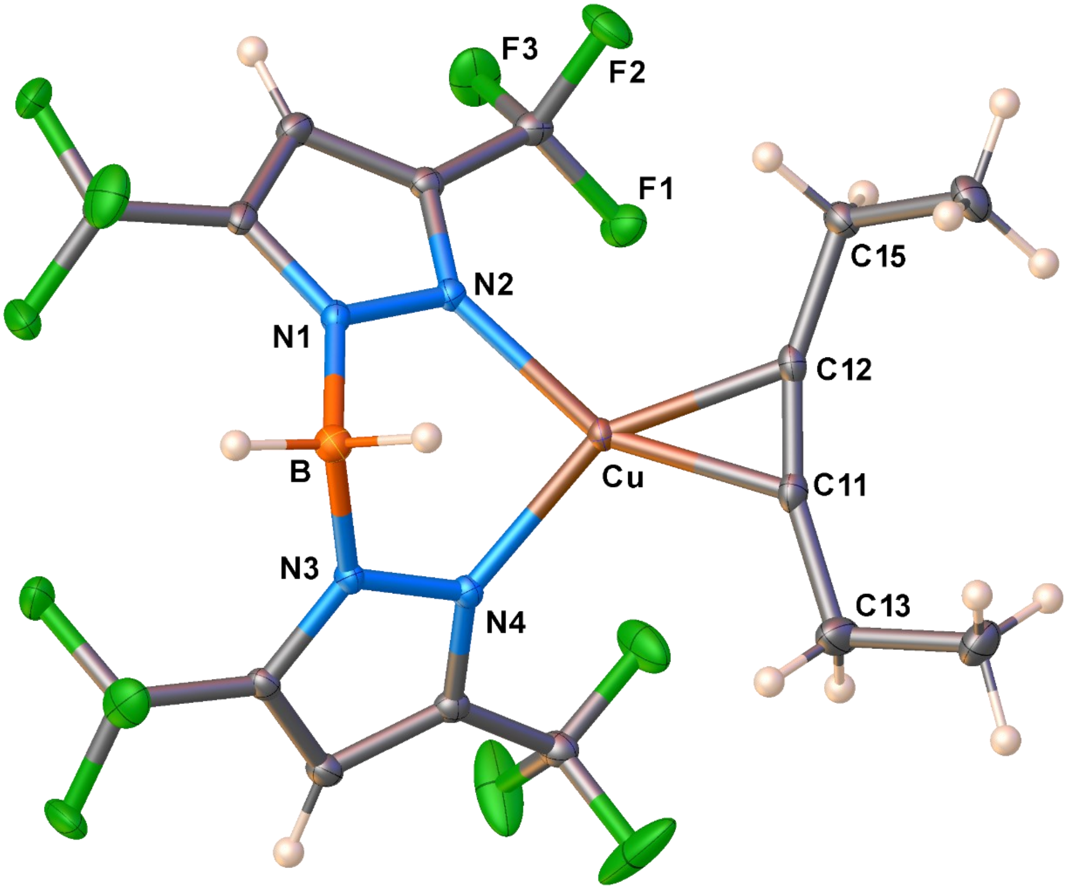

Figure S24:Molecular structure of [$H_2B(3,5\text{-}(CF_3)_2Pz)_2$]Cu(EtC≡CEt)(**6**).

Table S9: Crystal data and structure refinement for [$H_2B(3,5\text{-}(CF_3)_2Pz)_2$]Cu(EtC≡CEt)(**6**).

Identification code	rad769_0m_a
Empirical formula	$C_{16}H_{14}BCuF_{12}N_4$
Formula weight	564.66
Temperature/K	100.0
Crystal system	triclinic
Space group	P-1
a/Å	8.1718(4)
b/Å	11.6524(6)
c/Å	12.6430(7)
α/°	63.9860(10)

β/°	78.525(2)
γ/°	80.5110(10)
Volume/Å^3	1056.16(10)
Z	2
ρ_{calc}g/cm^3	1.776
μ/mm^{-1}	1.151
F(000)	560.0
Crystal size/mm^3	0.23 × 0.2 × 0.2
Radiation	MoKα (λ = 0.71073)
2Θ range for data collection/°	5.812 to 61.398
Index ranges	-11 ≤ h ≤ 11, -16 ≤ k ≤ 16, -18 ≤ l ≤ 18
Reflections collected	53722
Independent reflections	6553 [R_{int} = 0.0468, R_{sigma} = 0.0310]
Data/restraints/parameters	6553/0/318
Goodness-of-fit on F^2	1.018
Final R indexes [I>=2σ (I)]	R_1 = 0.0365, wR_2 = 0.0696
Final R indexes [all data]	R_1 = 0.0494, wR_2 = 0.0748
Largest diff. peak/hole / e Å^{-3}	0.70/-0.66

Table S10: Bond Lengths for [$H_2B(3,5-(CF_3)_2Pz)_2$]Cu(EtC≡CEt)(**6**).

Atom	Atom	Length/Å	Atom	Atom	Length/Å
Cu	N2	1.9992(14)	N2	C1	1.336(2)
Cu	N4	2.0022(14)	N3	N4	1.3553(19)
Cu	C11	1.9818(16)	N3	C8	1.354(2)
Cu	C12	1.9862(16)	N3	B	1.572(2)
F1	C4	1.338(2)	N4	C6	1.337(2)
F2	C4	1.3385(19)	C1	C2	1.391(2)
F3	C4	1.336(2)	C1	C4	1.488(2)
F4	C5	1.338(2)	C2	C3	1.378(2)
F5	C5	1.339(2)	C3	C5	1.495(2)
F6	C5	1.334(2)	C6	C7	1.393(2)
F7	C9	1.317(2)	C6	C9	1.486(2)
F8	C9	1.331(2)	C7	C8	1.376(2)
F9	C9	1.325(2)	C8	C10	1.494(2)
F10	C10	1.333(2)	C11	C12	1.235(2)
F11	C10	1.331(2)	C11	C13	1.480(3)

Table S10: Bond Lengths for [$H_2B(3,5-(CF_3)_2Pz)_2$]Cu(EtC≡CEt)(**6**).

Atom	Atom	Length/Å	Atom	Atom	Length/Å
F12	C10	1.340(2)	C12	C15	1.479(2)
N1	N2	1.3556(19)	C13	C14	1.519(3)
N1	C3	1.355(2)	C15	C16	1.530(2)
N1	B	1.574(2)			

Table S11: Bond Angles for [$H_2B(3,5-(CF_3)_2Pz)_2$]Cu(EtC≡CEt)(**6**).

Atom	Atom	Atom	Angle/°	Atom	Atom	Atom	Angle/°
N2	Cu	N4	91.94(6)	F5	C5	C3	109.60(15)
C11	Cu	N2	153.49(6)	F6	C5	F4	107.36(15)
C11	Cu	N4	114.02(7)	F6	C5	F5	107.02(14)
C11	Cu	C12	36.26(7)	F6	C5	C3	112.67(15)
C12	Cu	N2	117.63(6)	N4	C6	C7	111.25(15)
C12	Cu	N4	150.28(7)	N4	C6	C9	121.45(15)
N2	N1	B	118.63(13)	C7	C6	C9	127.20(15)
C3	N1	N2	108.64(14)	C8	C7	C6	103.56(14)
C3	N1	B	132.73(14)	N3	C8	C7	109.69(14)
N1	N2	Cu	114.97(10)	N3	C8	C10	123.16(15)
C1	N2	Cu	138.00(12)	C7	C8	C10	127.13(15)
C1	N2	N1	106.92(13)	F7	C9	F8	105.94(16)
N4	N3	B	117.90(13)	F7	C9	F9	108.08(17)
C8	N3	N4	108.79(13)	F7	C9	C6	113.30(15)
C8	N3	B	133.16(14)	F8	C9	C6	112.76(14)
N3	N4	Cu	115.60(10)	F9	C9	F8	105.75(15)
C6	N4	Cu	137.17(12)	F9	C9	C6	110.58(15)
C6	N4	N3	106.71(13)	F10	C10	F12	107.32(14)
N2	C1	C2	111.18(15)	F10	C10	C8	112.52(14)
N2	C1	C4	121.24(15)	F11	C10	F10	107.40(15)
C2	C1	C4	127.55(16)	F11	C10	F12	107.32(14)
C3	C2	C1	103.69(15)	F11	C10	C8	112.48(14)
N1	C3	C2	109.56(15)	F12	C10	C8	109.54(15)
N1	C3	C5	123.58(16)	C12	C11	Cu	72.06(11)
C2	C3	C5	126.86(16)	C12	C11	C13	162.90(17)
F1	C4	F2	106.64(14)	C13	C11	Cu	125.00(12)
F1	C4	C1	112.64(14)	C11	C12	Cu	71.68(10)

Table S11: Bond Angles for $[H_2B(3,5\text{-}(CF_3)_2Pz)_2]Cu(EtC{\equiv}CEt)$(**6**).

**Atom**	**Atom**	**Atom**	**Angle/°**	**Atom**	**Atom**	**Atom**	**Angle/°**
F2	C4	C1	112.12(14)	C11	C12	C15	160.33(17)
F3	C4	F1	107.19(14)	C15	C12	Cu	127.98(12)
F3	C4	F2	106.86(14)	C11	C13	C14	112.22(16)
F3	C4	C1	111.04(14)	C12	C15	C16	111.67(15)
F4	C5	F5	107.17(15)	N3	B	N1	106.45(13)
F4	C5	C3	112.71(15)				

Crystal Data for $C_{16}H_{14}BCuF_{12}N_4$ (M =564.66 g/mol): triclinic, space group P-1 (no. 2), a = 8.1718(4) Å, b = 11.6524(6) Å, c = 12.6430(7) Å, α = 63.9860(10)°, β = 78.525(2)°, γ = 80.5110(10)°, V = 1056.16(10) Å^3, Z = 2, T = 100.0 K, μ(MoKα) = 1.151 mm^{-1}, $Dcalc$ = 1.776 g/cm^3, 53722 reflections measured (5.812° ≤ 2Θ ≤ 61.398°), 6553 unique (R_{int} = 0.0468, R_{sigma} = 0.0310) which were used in all calculations. The final R_1 was 0.0365 (I > 2σ(I)) and wR_2 was 0.0748 (all data).

Raman Spectra of Free Alkynes

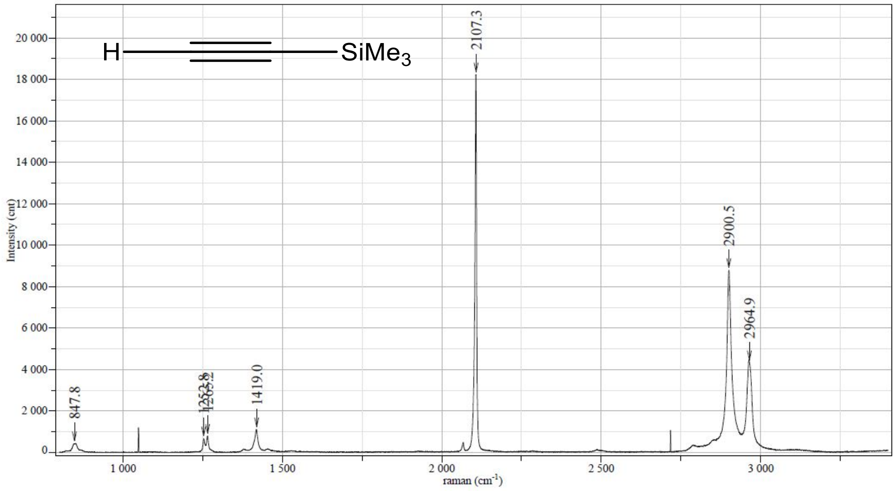

Exposition	5	Slit	100
Accumulation	1 x 21	Operator	Anurag
Laser	632.817	Sample	TMSacetylene
Spectro	Auto	Remark	
Hole	200	Power	

Figure S25: Raman spectrum of Trimethylsilylacetylene.

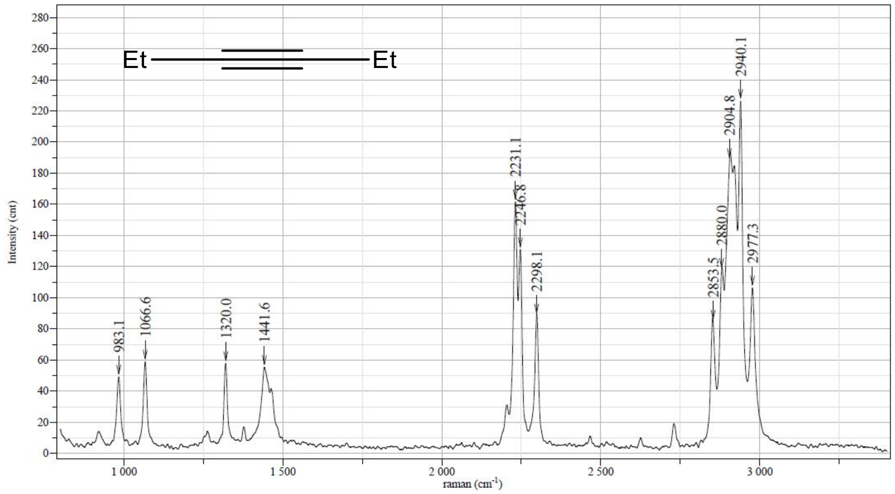

Exposition	1	Slit	100
Accumulation	1 x 21	Operator	Anurag
Laser	632.817	Sample	3-hexyne
Spectro	Auto	Remark	
Hole	200	Power	

Figure S26: Raman spectrum of 3-hexyne.

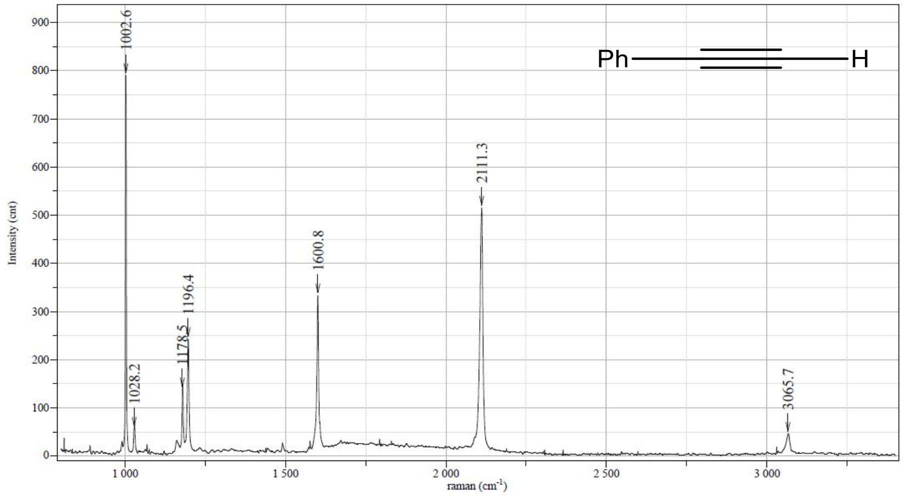

Exposition	5	Slit	100
Accumulation	10 x 4	Operator	
Laser	632.817	Sample	
Spectro	Multi	Remark	
Hole	200	Power	

Figure S27: Raman spectrum of Phenylacetylene.

Computational Details

Geometry optimizations and subsequent calculations were done using the ADF2019 code.[12,13] Triple-ξ and two polarization functions (STO-TZ2P) basis sets were employed within the generalized gradient approximation (GGA) according to the BP86 exchange-correlation functional and the empirical dispersion correction to DFT (DFT-D) given by the pair-wise Grimme correction (D3)[14-17] and Becke-Johnson damping functions.[18,19] Energy convergence criterion was set to 10^{-5} Hartree, gradient convergence criteria to 10^{-4} Hartree/Å, and radial convergence criteria to 10^{-3} Å, to achieve final relaxed structures. The energy decomposition analysis (EDA)[20-22] describes ΔE_{int} in terms of different meaningful quantities accounting for the electrostatic interaction (ΔE_{elstat}) between the defined fragments, the repulsive exchange (ΔE_{Pauli}) interaction owing to the four-electron/two-orbital repulsion between occupied orbitals from the different fragments. The orbital (covalent) interaction (ΔE_{orb}), which comes from the orbital relaxation and the orbital mixing between the fragments. Moreover, the dispersion interaction (ΔE_{disp}) was evaluated via the pairwise correction of Grimme[6](DFT-D3), denoting a stabilizing character. The counterpoise method was employed to overcome basis set superposition error (BSSE), denoting values lower than 2.0 kcal mol^{-1}. According to:

$$\Delta E_{int} = \Delta E_{Pauli} + \Delta E_{elstat} + \Delta E_{orb} + \Delta E_{disp}$$

Table S12. Energy decomposition analysis of the interaction energy for $(Ph_3P)_2Ni(HC{\equiv}CH)$ species accounting for the acetylene coordination. Values in kcal.mol^{-1}. Vibrational frequencies in cm^{-1}. Experimental value from [23].

ΔE_{Pauli}	172.1	
ΔE_{Elstat}	-132.1	53.2%
ΔE_{orb}	-107.8	43.4%
ΔE_{Disp}	-8.3	3.4%
ΔE_{int}	-75.9	
$\pi \rightarrow Ni$	-77.6	72.0%
$\sigma \leftarrow Ni$	-15.3	14.2%
$\nu_{C\equiv C}$		
Calc.	1641	
Exp.	1630	

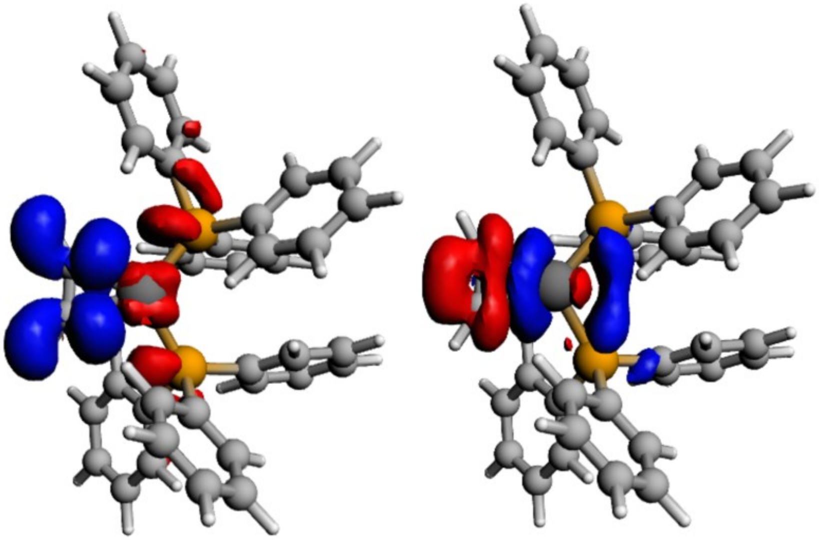

Figure S28. Deformation densities account for the π-backbonding (left) and σ-donation (right) contribution to the bonding scheme in the formation of $(Ph_3P)_2Ni(HC{\equiv}CH)$. Charge flow from red to blue.

References

1. Parasar, D.; Ponduru, T.T.; Noonikara-Poyil, A.; Jayaratna, N.B.; Dias, H.V.R. Acetylene and terminal alkyne complexes of copper(i) supported by fluorinated pyrazolates: syntheses, structures, and transformations. *Dalton Transactions* **2019**, *48*, 15782-15794, doi:10.1039/C9DT03350E.
2. Noonikara-Poyil, A.; Ridlen, S.G.; Dias, H.V.R. Isolable Copper(I) η2-Cyclopropene Complexes. *Inorganic Chemistry* **2020**, *59*, 17860-17865, doi:10.1021/acs.inorgchem.0c02886.
3. Parasar, D.; Almotawa, R.M.; Jayaratna, N.B.; Ceylan, Y.S.; Cundari, T.R.; Omary, M.A.; Dias, H.V.R. Synthesis, Photophysical Properties, and Computational Analysis of Di- and Tetranuclear Alkyne Complexes of Copper(I) Supported by a Highly Fluorinated Pyrazolate. *Organometallics* **2018**, *37*, 4105-4118, doi:10.1021/acs.organomet.8b00657.
4. Dias, H.V.R.; Richey, S.A.; Diyabalanage, H.V.K.; Thankamani, J. Copper(I) complexes supported by a heavily fluorinated bis(pyrazolyl)borate: syntheses and characterization of [H2B(3,5-(CF3)2Pz)2]CuL (where L = PPh3, NCCH3, HCCPh, H2CCHPh) and {[H2B(3,5-(CF3)2Pz)2]Cu}2(1,5-COD). *Journal of Organometallic Chemistry* **2005**, *690*, 1913-1922, doi:10.1016/j.jorganchem.2004.10.047.
5. Bhagavantam, S. Raman Spectra of Gases. *Nature* **1931**, *127*, 817-818, doi:10.1038/127817b0.
6. Thompson, J.S.; Whitney, J.F. Preparation and structural characterization of acetylene(2,2'-dipyridylamine)copper(I) tetrafluoroborate. *Journal of the American Chemical Society* **2002**, *105*, 5488-5490, doi:10.1021/ja00354a056.
7. Thompson, J.S.; Whitney, J.F. Copper(I) complexes with unsaturated small molecules. Preparation and structural characterization of copper(I)-di-2-pyridylamine complexes with olefins, acetylene, and carbon monoxide. *Inorganic Chemistry* **2002**, *23*, 2813-2819, doi:10.1021/ic00186a020.
8. Munakata, M.; Kitagawa, S.; Kawada, I.; Maekawa, M.; Shimono, H. Synthesis, formation constants and structures of ternary copper(I) complexes with 1,10-phenanthroline and alkynes. *Journal of the Chemical Society, Dalton Transactions* **1992**, 10.1039/dt9920002225, 2225, doi:10.1039/dt9920002225.
9. Chesnokov, G.A.; Topchiy, M.A.; Dzhevakov, P.B.; Gribanov, P.S.; Tukov, A.A.; Khrustalev, V.N.; Asachenko, A.F.; Nechaev, M.S. Eight-membered-ring diaminocarbenes bearing naphthalene moiety in the backbone: DFT studies, synthesis of amidinium salts, generation of free carbene, metal complexes, and solvent-free copper catalyzed azide–alkyne cycloaddition (CuAAC) reaction. *Dalton Transactions* **2017**, *46*, 4331-4345, doi:10.1039/c6dt04484k.
10. Fletcher, J.T.; Sobczyk, J.M.; Gwazdacz, S.C.; Blanck, A.J. Antimicrobial 1,3,4-trisubstituted-1,2,3-triazolium salts. *Bioorganic & Medicinal Chemistry Letters* **2018**, *28*, 3320-3323, doi:10.1016/j.bmcl.2018.09.011.
11. Kloss, F.; Köhn, U.; Jahn, B.O.; Hager, M.D.; Görls, H.; Schubert, U.S. Metal-Free 1,5-Regioselective Azide-Alkyne [3+2]-Cycloaddition. *Chemistry - An Asian Journal* **2011**, *6*, 2816-2824, doi:10.1002/asia.201100404.
12. te Velde, G.; Baerends, E.J. Precise density-functional method for periodic structures. *Physical Review B* **1991**, *44*, 7888-7903, doi:10.1103/PhysRevB.44.7888.

13. Fonseca Guerra, C.; Snijders, J.G.; te Velde, G.; Baerends, E.J. Towards an order- N DFT method. *Theoretical Chemistry Accounts: Theory, Computation, and Modeling (Theoretica Chimica Acta)* **1998**, *99*, 391-403, doi:10.1007/s002140050353.
14. Grimme, S. Accurate description of van der Waals complexes by density functional theory including empirical corrections. *Journal of Computational Chemistry* **2004**, *25*, 1463-1473, doi:10.1002/jcc.20078.
15. Grimme, S. Semiempirical GGA-type density functional constructed with a long-range dispersion correction. *Journal of Computational Chemistry* **2006**, *27*, 1787-1799, doi:10.1002/jcc.20495.
16. Grimme, S.; Antony, J.; Ehrlich, S.; Krieg, H. A consistent and accurate ab initio parametrization of density functional dispersion correction (DFT-D) for the 94 elements H-Pu. *The Journal of Chemical Physics* **2010**, *132*, 154104, doi:10.1063/1.3382344.
17. Grimme, S. Density functional theory with London dispersion corrections. *WIREs Computational Molecular Science* **2011**, *1*, 211-228, doi:10.1002/wcms.30.
18. Johnson, E.R.; Becke, A.D. A post-Hartree–Fock model of intermolecular interactions. *The Journal of Chemical Physics* **2005**, *123*, 024101, doi:10.1063/1.1949201.
19. Grimme, S.; Ehrlich, S.; Goerigk, L. Effect of the damping function in dispersion corrected density functional theory. *Journal of Computational Chemistry* **2011**, *32*, 1456-1465, doi:10.1002/jcc.21759.
20. Versluis, L.; Ziegler, T. The determination of molecular structures by density functional theory. The evaluation of analytical energy gradients by numerical integration. *The Journal of Chemical Physics* **1988**, *88*, 322-328, doi:10.1063/1.454603.
21. Ziegler, T.; Rauk, A. On the calculation of bonding energies by the Hartree Fock Slater method. *Theoretica Chimica Acta* **1977**, *46*, 1-10, doi:10.1007/bf00551648.
22. Hopffgarten, M.v.; Frenking, G. Energy decomposition analysis. *WIREs Computational Molecular Science* **2011**, *2*, 43-62, doi:10.1002/wcms.71.
23. Pörschke, K.R.; Tsay, Y.-H.; Krüger, C. Ethynebis(triphenylphosphane)nickel(0). *Angewandte Chemie International Edition in English* **1985**, *24*, 323-324, doi:10.1002/anie.198503231.
